# Unifying colors by primes

**DOI:** 10.1038/s41377-023-01073-x

**Published:** 2023-02-01

**Authors:** Han-Lin Li, Shu-Cherng Fang, Bertrand M. T. Lin, Way Kuo

**Affiliations:** 1 Department of Management Science, City University of Hong Kong, Hong Kong, China; 2 Department of Industrial and Systems Engineering, North Carolina State University, Raleigh, NC 27695 USA; 3 Institute of Information Management, Yang Ming Chiao Tung University, Taiwan, China; 4 Hong Kong Institute for Advanced Study, City University of Hong Kong, Hong Kong, China

**Keywords:** Optics and photonics, Optical techniques

## Abstract

RGB and CYMK are two major coloring schemes currently available for light colors and pigment colors, respectively. Both systems use letter-based color codes that require a large range of values to represent different colors. The problem is that these two systems are hard to use for manipulating any operations involving combinations of colors, and they lack the capacity for inter-changeability or unification. Based on prime number theory and Goldbach’s conjecture, this study presents a universal color system (C_235_) using a number-based structure to encode, compute and unify all colors on a color wheel. The proposed C_235_ system offers a unified representation for the efficient encoding and effective manipulation of color. It can be applied to designing a high-rate LCD system and colorizing objects with multiple attributes and DNA codons, opening the door to manipulating colors and lights for even broader applications.

## Abstract (web summary)

The C_235_ color system unifies RGB, CMYK and HSV wheels with high compression rates and low compression errors.

## Graphical abstract

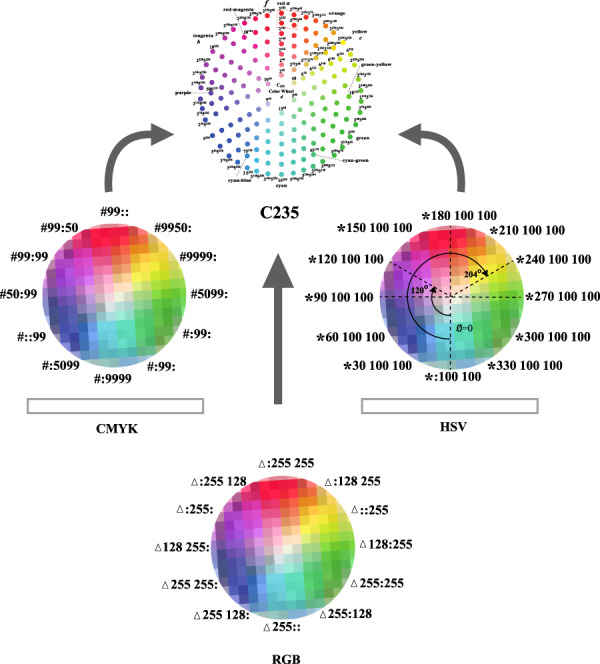

## Introduction

Numbers and colors are powerful tools for expressing objects such as people, goods, and DNA. The former can quantify objects and the latter can represent them visually. Isaac Newton’s theory of light claims that all colors can be generated from three basic colors: red, green, and blue^1^. Originating from Newton’s theory^1^, RGB (Red, Green, Blue), a light-color structure that contains 3 × 256 values of letter symbols, and CMYK (Cyan, Magenta, Yellow, Key black), a pigment-color structure that contains 4 × 100 values of letter symbols^2,3^, have become the most popular color frames used today. Most of the other color frames, such as HSV (Hue, Saturation, Value) are derived from RGB and CMYK^3^.

In the RGB frame, each of R, G, and B colors has 256 values expressed as [0, 1, 2,…, 255], and is coded as (*r*, *g*, *b*). In the CMYK frame, each of C, M, Y, and K has 100 values, expressed as [0, 1, 2,…, 99], and is coded as (*c*, *m*, *y*, *k*).

The weakness of the current CMYK and RGB frames are given below.

(i) Expression problems: R, G, B and C, M, Y, K are letter symbols; it is hard to use them to explicitly express the relationship between colors. Take the RGB framework as an example. Based on key colors R, G, and B, another nine colors {RY, Y, YG, GC, C, CB, BM, M, MR} can be deduced, where Y stands for yellow, C for cyan, and M for magenta. However, it is not easy for a user to directly realize the components of color from these letter symbols. Difficulties arise in various application contexts without a specific mechanism for mathematical operations. For instance, what is the complement color of R? What are the triad-complementary pairs within these 12 colors?
(ii) Computing problems: Letter symbols in the current color frames are hard to use for color computation. For instance, what is the resulting color after blending four colors of RY, GC, CB, and MR? Moreover, what is the reflecting color of an apple if we use a blue light to irradiate a green apple?
(iii) Unification problems: Letter symbols are hard to use for unifying pigment colors and light colors, same for unifying RGB, CMYK, and HSV frames together. Such issues may cause ineffective conversions among different colors^4^.
(iv) Size problems: In a CMYK frame, each of *c*, *m*, *y*, and *k* may assume 100 values, while in an RGB frame, each of *r*, *g*, and *b* may assume 256 values. Take RGB as an example. Each of the R, G, and B colors has 256 values, thus resulting in 3 × 256^2^ hues, which makes it challenging to distribute and allocate these many colors and hues on a color wheel^4^^,^^5^. In addition, the large number of color values may cause a huge computational burden for combining some of them to generate preferred colors^6^.

The weakness of RGB and CMYK is further reflected in emerging applications, such as cell phones, PCs, and TVs, in which RGB and CMYK are widely applied. For current technologies, each pixel on an LCD^5^ screen requires 3 × 8 = 24 pulses to generate R, G, and B lights^7^, which is both time- and energy-consuming. Moreover, the transformation between lights and colors is so complicated that no usable smart system is currently available^8,9^.

In this study, we present a new color framework based on the prime number theory^10,11^ and Goldbach’s conjecture^10,12^, referred to as C_235_, to encode colors and colorize objects. The aim is to solve bottlenecks inherent in existing methods. Prime number theory claims that any natural number larger than 1 can be uniquely expressed as the product of other prime numbers. This result is widely used for classifying information in the field of cryptography^13^ and optimization^14–16^. Goldbach’s conjecture further claims that any even number larger than 2 can be expressed as the sum of two prime numbers.

In our study, we choose the first three prime numbers 2, 3, and 5, to represent the three basic colors of red, green and blue, respectively. Since the white light is a combination of red, green, and blue lights, we may denote the white light by 30 = 2 × 3 × 5. Cyan is composed of green (3) and blue (5), so 3 × 5 = 15 is used to denote the cyan color. Similarly, 2 × 5 = 10 represents magenta, 2 × 3 = 6 represents yellow, and 15 × 10 × 6 = 30^2^ represents gray. The exponent of 30 is used to indicate the gray level. Therefore, 30^2^ is darker than 30^1^.

Furthermore, we can use seven numerical symbols to replace seven basic letter symbols, i.e., (2, 3, 5, 15, 10, 6, 30) = (R, G, B, C, M, Y, K) and another six numerical symbols for the corresponding letter symbols:

(2^2^ × 3 = 12, 3^2^ × 2 = 18, 3^2^ × 5 = 45, 5^2^ × 3 = 75, 5^2^ × 2 = 50, 2^2^ × 5 = 20) = (RY, YG, GC, CB, BM, MR)

Consequently, we can use 12 numbers (i.e., **2**, 12, 6, 18, **3**, 45, 15, 75, **5**, 50, 10, 20) to express 12 key hues, and use number 30 to express grayness. In addition, we can use $\left\langle {2^1,2^2, \ldots ,2^{256}} \right\rangle$, $\left\langle {3^1,3^2, \ldots ,3^{256}} \right\rangle$, and $\left\langle {5^1,5^2, \ldots ,5^{256}} \right\rangle$ to express the 256 levels of the basic colors R, G, and B, respectively. The mixtures of colors can then be efficiently expressed. For instance, the mixture of the basic colors R at level 10, G at level 20 and B at level 30 becomes

$$2^{10} \times 3^{20} \times 5^{30} = 5^{10} \times 15^{10} \times 30^{10} = 75^{10} \times 30^{10}$$

which is the color CB (75 for color cyan-blue) at level 10, adding gray (30 for grayness) at level 10.

Moreover, we can define that if the product of several colors equals an integer power of, say 30^*k*^ for *k* = 1, 2, …, then these colors are complementary. For instance, R, G, and B are complementary, since 2×3×5 = 30. Similarly, YG and BM are complementary since 18 × 50 = 900 = 30^2^.

Generally speaking, the proposed C_235_ color framework works much more efficient for encoding, computing, and unifying colors than the existing RGB and CMYK frames. By utilizing Goldbach’s conjecture, this study shows a novel way to compress the RGB color wheel into a much smaller C_235_ wheel, alleviating the size problem noted with the current RGB frame. Furthermore, we show that the proposed C_235_ color frame can be readily adopted for colorizing any objects with multiple attributes^14^, designing LCD light systems^5^, and coloring DNA codons^17^.

The rest of the paper is organized as follows: Section 2 describes two main results, namely, the C_235_ ring and the C_235_ wheel. Section 3 shows how to apply the proposed C_235_ system for designing a high-rate LCD light system. Section 4 discusses the methods and information involved in colorizing objects with multiple attributes^14,15^ and DNA codons^17,18^ using the proposed C_235_ system. Section 5 concludes the paper.

## Results

This study conveys two sets of major results. The first one is the development of a C_235_ system using the first three primes 2, 3, and 5 for colors R, G, and B, respectively, to generate color codes. We reasoned that when a large number of colors, say 256^3^, are involved in a system, then we really need to develop a compression mechanism that allocates the colors of concern on a color wheel. The second result indeed shows such a desired compression mechanism of the proposed C_235_ system for allocating all colors on layered rings for easy display and manipulation. The details are presented below.

### Result 1: Development of C_235_ system

A C_235_ color system represents colors R, G, and B by primes 2, 3, and 5, respectively. In this color frame, code <2> is for red color, <3> for green color, and <5> for blue color. Consequently, code <6> = <2 × 3> is for color yellow (Y), code <15> = <3 × 5> is for color cyan (C), code <18> = <3 × 6> is for color yellow-green (YG), and code <45> = <3 × 15> is for color cyan-green (CG). Color in the C_235_ system is also associated with a gray level for its lightness/thickness. Since <30> = <2 × 3 × 5> represents a white light, we use the powers of 30 (such as 30^1^, 30^2^, 30^3^, …) to indicate the grayness levels. The general rule is that higher power means a darker/thicker color.

**Fig. 1:**
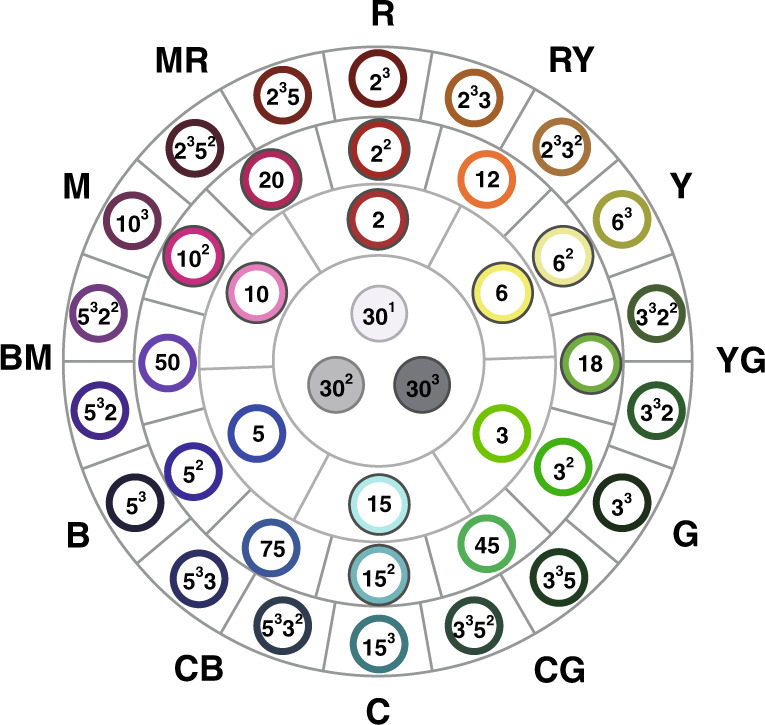
A C_235_ system with the first 36 hue codes in a ring form. The basic C_235_ color system has 36 hues/colors with 3 gray levels organized in 3 rings and 12 sectors – the inner circle has 3 gray codes surrounded by 36 hues/color codes spreading in 3 rings that belong to the 12 sectors outside the big circle

Figure 1 shows a basic C_235_ color system of 36 hues/colors with three gray levels organized in three rings and 12 sectors—the inner circle has three gray codes (such as 30^1^, 30^2^, and 30^3^, ⋯) surrounded by 36 hues/color codes (such as 2, 3, 5, 6, 12, 3^2^, and 2^2^5^3^) spreading in three rings that belong to the 12 sectors (such as R, Y, G, RY, and YG) outside the big circle. This C_235_ system makes plotting a specific color more convenient. For instance, <2 × 30> = <2 × 2 × 3 × 5> represents a color composed of hue <2> located in the first ring with a gray level <30> that belongs to the R sector. Hence it is “light red”. For another instance, <2 × 5^2^ × 30^3^> is a color composed of hue <50 = 2 × 5^2^> and a gray level <30^3^> . Noting that hue <50> is located at ring 2 of the BM sector, we know that the color is a dark blue magenta with more blue than red.

The steps for building the C_235_ system are described as follows.

Step 1: Choose the smallest three prime numbers 2, 3, and 5 to denote three basic colors. Since the red light, green light, and blue light are irreducible, we denote 2, 3, and 5 as red, green, and blue, respectively. Because the white light is a combination of red, green, and blue lights, we denote 30 = 2×3×5 as the white and gray light. Then, we use 3 × 5 = 15 to denote the cyan color, since cyan is composed of green (3) and blue (5). Similarly, we use 2 × 5 = 10 to represent the magenta color, and use 2 × 3 = 6 to represent the yellow color, and use 15 × 10 × 6 = 30^2^ to represent gray color. Table 1 explains more about this coding framework.

**Table 1:** A C_235_ system with the first 36 hue codes in a table form

Sector name & code	Contained hue code	Name of hue	Sector name and code	Contained hue code	Name of hue
R <2>	<2> <2^2^> <2^3^>	red 1 red 2 red 3	C <15>	<15> <15^2^> <15^3^>	cyan 1 cyan 2 cyan 3
RY (orange) <12>	<2^2^ × 3> <2^3^ × 3> <2^3^ × 3^2^>	red 2 - green red 3 - green red 3 - green 2	CB <75>	<3 × 5^2^> <3 × 5^3^> <3^2^ × 5^3^>	green - blue 2 green - blue 3 green 2 - blue 3
Y <6>	<6> <6^2^> <6^3^>	yellow 1 yellow 2 yellow 3	B <5>	<5> <5^2^> <5^3^>	blue 1 blue 2 blue 3
YG <18>	<2 × 3^2^> <2 × 3^3^> <2^2^ × 3^3^>	red - green 2 red - green 3 red 2 - green 3	BM <50>	<2 × 5^2^> <2 × 5^3^> <2^2^ × 5^3^>	red - blue 2 red - blue 3 red 2 - blue 3
G <3>	<3> <3^2^> <3^3^>	green 1 green 2 green 3	M <10>	<10> <10^2^> <10^3^>	magenta 1 magenta 2 magenta 3
GC <45>	<3^2^ × 5> <3^3^ × 5> <3^3^ × 5^2^>	green 2 - blue green 3 - blue green 3 - blue 2	MR (purple) <20>	<2^2^ × 5> <2^3^ × 5> <2^3^ × 5^2^>	red 2 - blue red 3 - blue red 3 - blue 2

Step 2: Express pigment-color values based on Goldbach’s conjecture. Let *S* be the set composed of 0 and the first 18 prime numbers, i.e.,

$$S = \left\{ {0,\,2,\,3,\,5,\,7,\,11,\,13,\,17,\,19,\,23,\,29,\,31,\,37,\,41,\,43,\,47,\,53,\,59,\,61} \right\}$$

Then, we know from Goldbach’s results that any even number between 4 and 99 can be expressed as the sum of the two numbers in *S*; and any odd number between 0 and 99 can be expressed as the sum of the two numbers in *S* plus 1. This means that by using 4 × 18 = 72 values (together with 99 values of lightness), we can express all CMYK colors.

Step 3: Let <*i*> be the code of color *i*. We use codes $\left\langle 2 \right\rangle$, $\left\langle 3 \right\rangle$, $\left\langle 5 \right\rangle$, $\left\langle 6 \right\rangle$, $\left\langle {10} \right\rangle$, $\left\langle {15} \right\rangle$, and $\left\langle {30} \right\rangle$ to represent the seven key colors R (red), G (green), B (blue), C (cyan), M (magenta), Y (yellow), and W (white or gray). The code of color *i* is denoted as *Code*(*i*):

1 $$\begin{array}{l}{\it{{\mathrm{Code}}}}({\it{i}}) = \left\langle {2^{{{{\boldsymbol{r}}}}_{{{\boldsymbol{i}}}}}3^{{{{\boldsymbol{g}}}}_{{{\boldsymbol{i}}}}}5^{{{{\boldsymbol{b}}}}_{{{\boldsymbol{i}}}}}} \right\rangle = \left\langle {2^{100 - {{{\boldsymbol{c}}}}_{{{\boldsymbol{i}}}}}3^{100 - {{{\boldsymbol{m}}}}_{{{\boldsymbol{i}}}}}5^{100 - {{{\boldsymbol{y}}}}_{{{\boldsymbol{i}}}}}} \right\rangle ^{2.56}\\ = \left\langle {2^{{{{\boldsymbol{r}}}}_{{{\boldsymbol{i}}}} - {{{\boldsymbol{l}}}}_{{{\boldsymbol{i}}}}}3^{{{{\boldsymbol{g}}}}_{{{\boldsymbol{i}}}} - {{{\boldsymbol{l}}}}_{{{\boldsymbol{i}}}}}5^{{{{\boldsymbol{b}}}}_{{{\boldsymbol{i}}}} - {{{\boldsymbol{l}}}}_{{{\boldsymbol{i}}}}}30^{{{{\boldsymbol{l}}}}_{{{\boldsymbol{i}}}}}} \right\rangle \end{array}$$

where *l*_*i*_ = min{$r_i,g_i,b_i$}. We call <$2^{r_i - l_i}3^{g_i - l_i}5^{b_i - l_i}$> the hue code and <$30^{l_i}$> the gray code. We can also assign a unique identifier to *Code*(*i*) as

2 $$\begin{array}{l}{\it{ID}}\left( {\it{i}} \right) = 256^2\left( {{\it{r}}_{\it{i}}} \right) + 256\left( {{\it{g}}_{\it{i}}} \right) + {\it{b}}_{\it{i}}\\ \qquad\qquad\cong 16,777,215 - 167116.8{{{\boldsymbol{c}}}}_{{{\boldsymbol{i}}}} - 652.8{\it{m}}_{\it{i}} - 2.55{\it{y}}_{\it{i}}\end{array}$$

where *ID*(*i*) is an integer.

The key merit of Expressions (1) and (2) is that both pigment colors and light colors can be unified in the C_235_ frame. This renders potential usages of free color conversion between RGB and CMYK systems. In the meantime, Expression (1) and Expression (2) can also unify the RGB and CMYK frames, thus inducing their potential use in color conversions between RGB and CMYK frames.

Step 4: The idea of the merger of light color with pigment color is expressed using the following example. Suppose there is an incident light $\left\langle {2^{{{{\mathrm{\alpha }}}}_2}3^{{{{\mathrm{\alpha }}}}_3}5^{{{{\mathrm{\alpha }}}}_5}} \right\rangle$ and a piece of cellophane with color $\left\langle {2^{{{{\mathrm{\beta }}}}_2}3^{{{{\mathrm{\beta }}}}_3}5^{{{{\mathrm{\beta }}}}_5}} \right\rangle$. Let $\left\langle {2^{\sigma _2}3^{\sigma _3}5^{\sigma _5}} \right\rangle$ be the reflecting light using the incident light to irradiate the cellophane. Then, the formulae of $\sigma _k$ (for *k* = 2, 3, 5) are expressed as

$$\sigma _k = \frac{{{{{\mathrm{\alpha }}}}_k{{{\mathrm{\beta }}}}_{{{\mathrm{k}}}}}}{{256}}$$, for *k* = 2, 3, 5.

- Suppose $\alpha _k = 0$or $\beta _k = 0,\,{{{\mathrm{then}}}}\,\sigma _k = 0$ for *k* = 2, 3, 5.
- Suppose $$\beta _k = 256,\,{{{\mathrm{then}}}}\,\sigma _k = \alpha _{k.}$$
- Suppose the cellophane is red (such as $\beta _2 = 100,\,\beta _3 = \beta _5 = 0$) and the incident light is also red (such as ${{{\mathrm{\alpha }}}}_2 = 100,\,{{{\mathrm{\alpha }}}}_3 = {{{\mathrm{\alpha }}}}_5 = 0$), then the reflection light is pale red with $\sigma _2 = 39,\,\sigma _3 = \sigma _5 = 0$.
- Suppose the cellophane is yellow (such as ${{{\mathrm{\beta }}}}_2 = {{{\mathrm{\beta }}}}_3 = 50,\,{{{\mathrm{\beta }}}}_5 = 0$)and the incident light is red (i.e., ${{{\mathrm{\alpha }}}}_2 = {{{\mathrm{\alpha }}}}_3 = 50,\,{{{\mathrm{\alpha }}}}_5 = 0$), then the reflected light is weak bright red with $\sigma _2 = 20,\,\sigma _3 = \sigma _5 = 0$.

Step 5: The merger of colors can be operated as follows. Suppose that *i* and *j* are two light colors. Denote by *l* the merger of colors *i* and *j*. Then, we have

$$\left\langle {2^{r_l}3^{g_l}5^{b_l}} \right\rangle = \left\langle {2^{r_i + r_j}3^{g_i + g_j}5^{b_i + b_j}} \right\rangle$$

Step 6: The complements of colors can be expressed as follows.

Two colors $\left\langle {2^{r_i}3^{g_i}5^{b_i}} \right\rangle$ and $\left\langle {2^{r_j}3^{g_j}5^{b_j}} \right\rangle$ are complementary if $r_i + r_j = g_i + g_j = b_i + b_j$. Three colors $\left\langle {2^{r_i}3^{g_i}5^{b_i}} \right\rangle$, $\left\langle {2^{r_j}3^{g_j}5^{b_j}} \right\rangle$ and $\left\langle {2^{r_l}3^{g_l}5^{b_l}} \right\rangle$ are triad-complementary if $r_i + r_j + r_l = g_i + g_j + g_l = b_i + b_j + b_l$.

Step 7: The colors in HSV can be denoted as ($h_i,\,s_i,\,v_i$), where *h*, *s*, and *v* represent hue, saturation, and value, respectively. HSV is also a light-color frame that is highly related to RGB. To unify HSV by C_235_, we first convert ($h_i,\,s_i,\,v_i$) to $\left\langle {2^{255 - r_i}3^{255 - g_i}5^{255 - b_i}} \right\rangle$, and then convert it to $\left\langle {2^{\alpha _i}3^{\beta _i}5^{\sigma _i}} \right\rangle$.

Expressions (1) and (2) are based on an 8-bit color frame, where $c_i,\,m_i,\,y_i,\,k_i$∈ {0, 1, …, 99} and $r_i,\,g_i,\,b_i$ ∈ {0, 1, …, 255}. It can also be extended to a “high color” system with a 9-bit color frame, where $c_i,\,m_i,\,y_i,\,k_i$∈ {0, 1, …, 198} and $r_i,\,g_i,\,b_i$ ∈ {0, 1, …, 510}. For simplicity, this study considers only the 8-bit frame.

Once a ${{{\mathrm{C}}}}_{235}$ color system is built, we can easily generate color codes and manipulate color operations to answer related questions.

**Example 1**: Consider two pigment colors, *i* and *j* with $\left( {c_i,m_i,y_i,k_i} \right) = \left( {56,\,28,\,28,\,0} \right)$ and $\left( {c_j,m_j,y_j,k_j} \right) = \left( {39,\,0,\,0,\,28} \right)$. What are the universal codes, ID, and RGB codes of these two colors; and what color is created by emerging these two colors?

For color *i*, we have ${\mathrm{Code}}\left( i \right) = \left\langle {2^{100 - 56}3^{100 - 28}5^{100 - 28}} \right\rangle ^{2.56} = \left\langle {2^{r_i}3^{g_i}5^{b_i}} \right\rangle$. Therefore, $\left( {r_i,g_i,b_i} \right) = \left( {112,\,183,\,183} \right)$ and $ID\left( i \right) = 256^2 \times 112 + 256 \times 184 + 184 = 7,387,063$. For color *j*, we first convert$\left( {39,\,0,\,0,\,28} \right)$ into$\left( {67,\,28,\,28,\,0} \right)$. Therefore, $\left( {r_j,g_j,b_j} \right) = \left( {83,\,183,\,183} \right)$ and $ID\left( i \right) = 256^2 \times 83 + 256 \times 183 + 183 = 5,486,519$. Let color *l* be the merger of color *i* and color *j*, then we have *Code*(*l*) = $\left\langle {2^{196}3^{366}5^{366}} \right\rangle$, which can cause an overflow problem. Therefore, we adjust it as *Code*(*l*) = $\left\langle {2^{137}3^{256}5^{256}} \right\rangle ^{1.43}$.

**Example 2:** Where to find the three colors of Example 1 in the ring form of a C_235_ system?

$$\begin{array}{l} {{{{\mathrm{Since}}}}\,{\mathrm{Code}}(i)} = {\left\langle {2^{112}3^{183}5^{183}} \right\rangle } = {\left\langle {15^{71}30^{112}} \right\rangle } \\ {{\mathrm{Code}}(j)} = {\left\langle {2^{83}3^{183}5^{183}} \right\rangle }= {\left\langle {15^{100}30^{83}} \right\rangle } \\ {{\mathrm{Code}}(l)} = {\left\langle {2^{196}3^{366}5^{366}} \right\rangle } = {\left\langle {15^{170}30^{196}} \right\rangle }\end{array}$$

all three colors belong to sector C (15 for color cyan), with color *i* being located at ring 71, color *j* at ring 100, and color *l* at ring 170. Also, we know color *l* is darker than color *j* than color *i*.

Compared to the current RGB and CYMK color codes, the merits of C_235_ color frame are summarized below:

(i) A basic C_235_ color system can code and allocate up to 256^3^ colors on a two-dimensional disk.
(ii) The C_235_ color coding frame can unify the RGB and CMYK frames and provide easy conversion between different color frames.
(iii) The number-based C_235_ color frame allows easy manipulation of various color operations.

### Result 2: C_235_ color wheel (compression of large C_235_ system)

Color wheel is a powerful tool for displaying and manipulating colors^2,3^. However, the RGB wheel and CMYK wheel are not effective enough for the following reasons: Firstly, an RGB wheel contains 3 × 256^2^ color hues and a CMYK wheel contains 3 × 100^2^ hues. The number of hues is too large for manipulating or displaying colors^6^. Secondly, CMYK and RGB need 3 × 256 and 4 × 100 values, respectively, for representing colors. Thirdly, the CMYK wheel and RGB wheel are not interchangeable. This study, therefore, designs a compressed color wheel, called C_235_ wheel, which may integrate CMYK and RGB wheels with a compression error rate of less than 1.2%.

**Fig. 2:**
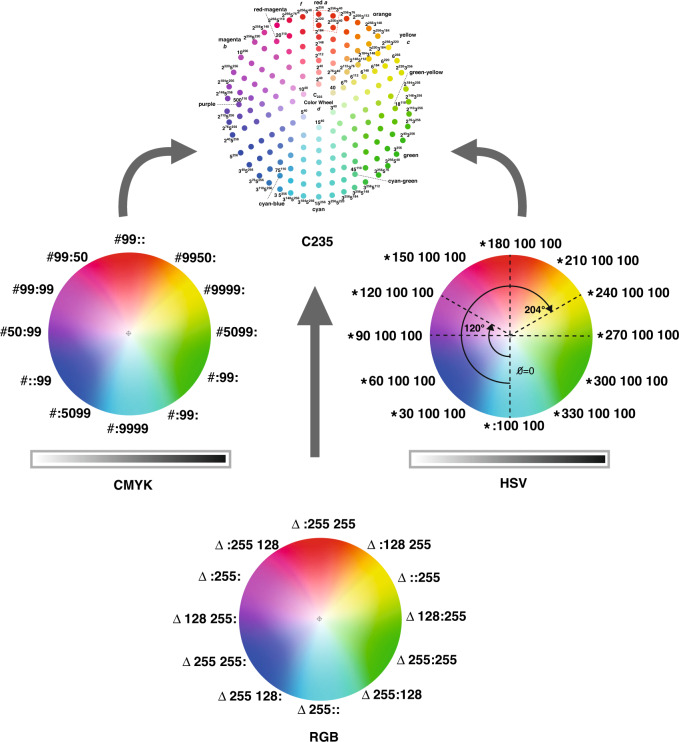
C_235_ wheel of unifying RGB, CMYK, and HSV. The compressed C_235_ wheel unifies CMYK, RGB, and HSV color systems using a much smaller number of raw colors (key values) to represent all 256^3^ colors

The compressed C_235_ wheel is designed to unify CMYK, RGB, and HSV color systems using a much smaller number of raw colors (key values) to represent all 256^3^ colors, as illustrated in Fig. 2. It has a high compression rate and low compression errors. Note that an RGB wheel^2,3^ contains 3 × 256^2^ color hues using 768 (= 3 × 256) key values, while a CMYK wheel contains 3 × 100^2^ hues using 400 (= 4 × 100) key values (Table 2).

**Table 2:** Comparison of C_235_ with other color frames

	Number of values	Number of hue blocks on a color wheel
CMYK	4 × 100 = 400	3 × 100^2^ = 30,000
RGB	3 × 256 = 768	3 × 256^2^ = 196,608
HSV	2 × 100 + 360 = 560	3 × 100^2^ = 30,000
*C*_235_	3 × 33 = 99	3 × 42^2^ = 5292

**Fig. 3:**
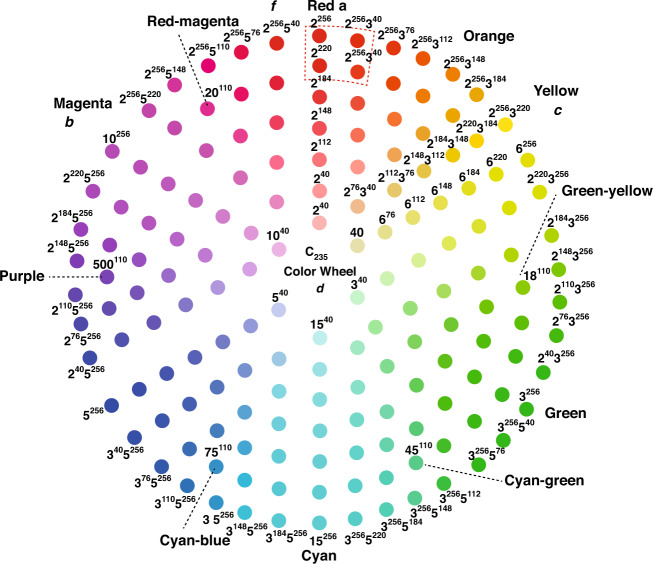
A compressed C_235_ wheel. The compression rate of C_235_ color wheel versus RGB wheel is about 37 and the compression error is within 1.2%. With a compressed C_235_ wheel, we can easily manipulate color operations

**Fig. 4:**
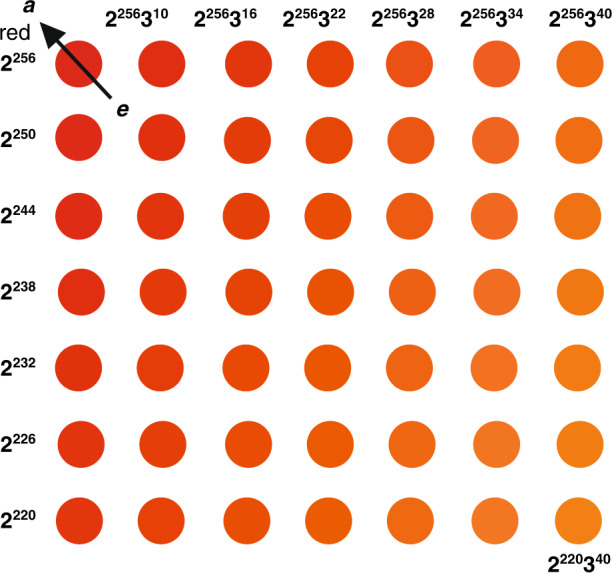
Zoom-in area in the compressed C_235_ wheel. ${\mathrm{Color}}\left( a \right) = \left\langle {2^{256}} \right\rangle ,\,{\mathrm{Color}}\left( b \right) = \left\langle {3^{256}} \right\rangle ,\,{\mathrm{Color}}\left( c \right) = \left\langle {5^{256}} \right\rangle ,{\mathrm{Color}}\left( d \right) = \left\langle {30^{256}} \right\rangle ,\,{\mathrm{Color}}\left( e \right) \cong \left\langle {2^{256}} \right\rangle ,\,{\mathrm{Color}}\left( f \right) \cong \left\langle {2^{256}} \right\rangle$

By performing the next five tasks, we can construct a compressed C_235_ color wheel (as shown in Fig. 3) that contains 3 × 42^2^ hues (42 is the maximum integer of *h* satisfying 6 × *h* ≤ 256) using only 99 key values. The compression rate of C_235_ color wheel versus the RGB wheel is $256^2/42^2 \cong 37$ and the compression error rate is within 1.2%. The graph of a C_235_ wheel is shown in Fig. 3. Zoom-in view of the dashed is depicted in Fig. 4.

The design of a C_235_ color wheel can be used in the following tasks:

Task 1: (33 Primes) Express pigment-color values based on Goldbach’s conjecture. Let *T* be the set composed of 0 and the first 33 prime numbers, i.e.,

$$\begin{array}{l}T = \left\{ {0,\,2,\,3,\,5,\,7,\,11,\,13,\,17,\,19,\,23,\,29,\,31,\,37,\,41,\,43,\,47,\,53,\,59,\,61,\,67,\,71,} \right.\\ \quad \quad \quad \left. {73,\,79,\,83,\,89,\,97,\,101,\,103,\,107,\,109,\,113,\,131,\,137,\,139} \right\}\end{array}$$

Then, we know that any even number between 4 and 255 can be expressed as the sum of the two numbers (repetitions allowed) in *T*; and any odd number between 0 and 255 can be expressed as the sum of the two numbers in *T* plus one. This means that we can represent all RGB colors using 3 × 33 = 99 key values.

Task 2: (Reduction of primes) Form a subset $U \subset T$, where *T* was obtained in Task 1

Subset *U* consists of 18 prime numbers as follows

$$U = \left\{ {5,\,11,\,17,\,23,\,29,\,41,\,47,\,53,\,59,\,71,\,83,\,89,\,101,\,107,\,113,\,127,\,131,\,137} \right\}$$

where the gap between two neighboring numbers is either 6 or 12. We can then express $r_i,\,g_i,\,b_i$ as

$$\begin{array}{*{20}{l}} {r_i} \hfill & = \hfill & {4 + f_1 + f_2} \hfill \\ {g_i} \hfill & = \hfill & {4 + f_3 + f_4} \hfill \\ {b_i} \hfill & = \hfill & {4 + f_5 + f_6} \hfill \end{array}$$

for $$f_1,f_2,f_3,f_4,f_5,f_6 \in U$$ and

$$f_1 + f_2,f_3 + f_4,f_5 + f_6 \in \left\{ {6h:h = 1,\,2, \ldots ,\,42} \right\}$$

Task 3: (Attractors) Generate attractors for the compressed C_235_ color wheel.

There are 3 × 42^2^ = 5292 attractors, each of which is expressed as $2^\alpha 3^\beta ,\,3^\beta 5^\sigma ,\,{{{\mathrm{and}}}}\,2^\alpha 5^\sigma$, where $\alpha ,\beta ,\sigma \in \left\{ {6h:h = 1,\,2, \ldots ,\,42} \right\} = \left\{ {4,\,6,\,10,\,16,\,22,\,28, \ldots ,\,256} \right\}$.

Denote *w-*Code(*i*) as the color code of an attractor *i* on a color wheel, expressed as

3 $$w - {\mathrm{Code}}\left( i \right) = \left\langle {2^{{\it{\alpha }} - \ell }3^{{\it{\beta }} - \ell }5^{{\it{\sigma }} - \ell }30^\ell } \right\rangle$$

where $$\ell = {{{\mathrm{min}}}}\left\{ {\alpha ,\beta ,\sigma } \right\}$$ and $$\alpha ,\beta ,\sigma \in \left\{ {4,6,10,16,22,28, \ldots ,256} \right\}$$.

Task 4: (Attractor assignment) Assign each of $\left\langle {2^{r_i}3^{g_i}5^{b_i}} \right\rangle$ and/or $\left\langle {2^{100 - r_i}3^{100 - g_i}5^{100 - b_i}} \right\rangle ^{2.56}$ to an attractor, where $r_i,\,g_i$, and $b_i$ are the closest integer values of ${{{\mathrm{\alpha }}}},\,{{{\mathrm{\beta }}}},\,{{{\mathrm{and}}}}\,{{{\mathrm{\sigma }}}}$, respectively. Four neighboring attractors form a block. There are 3 × 42^2^ = 5292 blocks on a C_235_ wheel.

Task 5: (Compression rate and error) The compression rate of the C_235_ color wheel versus the RGB wheel is $256^2/42^2 \cong 37$. The compression error can be computed here.

Because the distance from the center of a block to the 4 attractors of the block is bounded by $\left( {3^2 + 3^2} \right)^{0.5}$, the compression error rate is bounded from above by

$$((3^2 + 3^2)/(256^2 + 256^2))^{0.5} = 0.012$$

Notice that Expression (3) indicates that the C_235_ wheel uses a much smaller number of hue blocks to express codes than RGB and CMYK^2,3^ do. The comparison is listed in Table 2. Hence the C_235_ wheel is more effective in unifying colors than RGB, CMYK, and HSV are, as illustrated in Fig. 2. With a compressed C_235_ wheel, as shown in Fig. 3, we can easily manipulate color operations. For example, let *a*, *b*, *c*, *d*, *e*, and *f* be six reference points on the wheel. The dash-line enclosed area located at the top of the wheel is zoomed in as shown in the square under the wheel (Fig. 4). Several observations can be done here:

(i) Each color is expressed in a universal format that can be converted into an RGB, CMYK, or HSV frame. For instance, given $a = \left\langle {2^{256}} \right\rangle$, we have
  $$\left( {c_a,m_a,y_a,k_a} \right) = (100,\,0,\,0,\,0)$$
  $$\left( {h_a,s_a,v_a} \right) = (180^\circ ,100,\,100)$$
  For ${\mathrm{Color}}\left( b \right) = \left\langle {3^{256}} \right\rangle$, ${\mathrm{Color}}\left( c \right) = \left\langle {5^{256}} \right\rangle$, we can derive the same RGB and CMYK codes of colors *b* and *c*.
(ii) ${\mathrm{{Color}}}\left( d \right)$, located at the center of the wheel, is the merger of *a*, *b*, and *c*. The code is ${\mathrm{Color}}\left( d \right) = \left\langle {2^{256}} \right\rangle {{{\mathrm{merge}}}}\left\langle {3^{256}} \right\rangle {{{\mathrm{merge}}}}\left\langle {5^{256}} \right\rangle = \left\langle {30^{256}} \right\rangle$
(iii) ${\mathrm{Color}}(e) = \left\langle {2^{254}3^5} \right\rangle$, as shown in Fig.4, can be assigned to a neighboring attractor color ${\mathrm{Color}}\left( a \right)$ since $\left\langle {2^{254}3^5} \right\rangle \cong \left\langle {2^{256}} \right\rangle$.

${\mathrm{Color}}\left( f \right)$ is an overflow color denoted as ${\mathrm{Color}}\left( f \right) = \left\langle {2^{300}3^5} \right\rangle$. It can be rewritten as ${\mathrm{Color}}(f) = \left\langle {2^{256}3^{10}} \right\rangle ^{1.17} \cong \left\langle {2^{256}} \right\rangle ^{1.17}$, as shown at the top of the C_235_ wheel, and assigned to attractor point *a*. Moreover, the value 1.17 reflects that ${\mathrm{Color}}\left( f \right)$ is an overflow color.

## Discussion

### LCD light design using C_235_ system

**Fig. 5:**
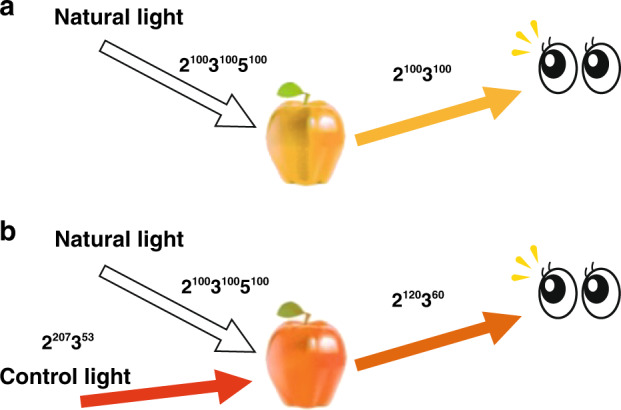
The design of a smart lighting system. **a** An apple observed under natural light. **b** An apple observed under a control light

LCD (Liquid Crystal Display) technology is widely adopted for making cellular phones, tablets, TVs, and many other electronic products. Most such involved displays use LED as the light source. A typical LED is fed with pulsed high currents for a short period of time using the pulse width modulation (PWM) technique to create modulated electronic pulses of the desired width^7^. Interestingly, the C_235_ color system allows users to conveniently merge lights and colors and facilitates the design of smart lighting systems by adjusting users’ preferences. In fact, such a system can be widely used in fashion shows, painting exhibitions, and commodity displays^19^. Suppose there is a natural light $2^{a_2}3^{a_3}5^{a_5}$ irradiating on an apple. The reflecting light $2^{b_2}3^{b_3}5^{b_5}$ is the apparent color of the apple. By adding an additional light $2^{c_2}3^{c_3}5^{c_5}$ to irradiate this apple, a preferred color can be visualized, as illustrated in Fig. 5.

A user will face decisions under different scenarios. Suppose a natural light $2^{a_2}3^{a_3}5^{a_5}$ is irradiating an apple. The apple’s reflecting light $2^{b_2}3^{b_3}5^{b_5}$ is the color of that apple. Now we add an additional light $2^{c_2}3^{c_3}5^{c_5}$ (the control light) to irradiate this apple. When we add a control light $2^{c_2}3^{c_3}5^{c_5}$ to irradiate the apple for a resulting color $2^{d_2}3^{d_3}5^{d_5}$, then the light-color transformation formula is

$$d_k = \left( {c_k + a_k} \right)b_k/256,\,{{{\mathrm{for}}}}\,k = 2,\,3,\,5$$

Assume that a yellow apple is irradiated by a white light, as shown in Fig. 5a. Then we have *a*_2_ = *a*_3_ = *a*_5_ = 100 and *b*_2_ = 100, *b*_3_ = 100, *b*_5_ = 0. Suppose that we want the apple to show the orange color (i.e., *d*_2_ = 120, *d*_3_ = 60, *d*_5_ = 0), then we know 120 = (*c*_2_ + 100)100/256 to get *c*_2_ ≐ 207. Similarly, 60 = (*c*_3_ + 100)100/256 implies that *c*_3_ ≐ 53. Therefore, the control light should be <2^207^3^53^>, as shown in Fig. 5b.

The present study is related to the design of PWM, especially for LCD. Consider the following application scenario.

**Example 3:** When a user wants to let an LCD pixel emit color $\left\langle {2^{224}3^{206}5^{102}} \right\rangle$, then what kind of pulse width do a traditional PWM and a ${{{\mathrm{C}}}}_{235}$ PWM need to generate?

A traditional PWM modulator^7,9^ demands more logical bits for representing colors and a sophisticated hardware design, as well as higher power consumption for emitting color lights. Please refer to Supplementary B for the technical details of the traditional PWM. The design proposed in this study is accomplished by employing an extended Goldbach conjecture in a C_235_ color system such that the nine pulse widths used for presenting each of R, G, or B at a pixel in the current 9-bit LCD display can be reduced to only two widths. Hence the new design may significantly reduce the technological complexity in associated devices.

One promising application of our color coding is for the LCD industry. Currently, to emit the R, G, and B lights of 256 colors, each pixel on the LCD screen has three LEDs under the control of a pixel circuit. All pixel circuits follow the order of an LCD central computing center^14^. In the current LCD system, for any integer *X* between 0 and 256, *X* can be expressed as

$$X = 128{{{\mathrm{T}}}}_{128} + 64{{{\mathrm{T}}}}_{64} + 32{{{\mathrm{T}}}}_{32} + 16{{{\mathrm{T}}}}_{16} + 8{{{\mathrm{T}}}}_8 + 4{{{\mathrm{T}}}}_4 + 2{{{\mathrm{T}}}}_2 = {{{\mathrm{T}}}}_1$$

where ${{{\mathrm{T}}}}_{128}$, ${{{\mathrm{T}}}}_{64}$,…, ${{{\mathrm{T}}}}_1$ are binary variables. The central computing center will order each R, G, and B LEDs of a pixel to generate up to eight pulse widths (${{{\mathrm{i}}}}.{{{\mathrm{e}}}}.,$ ${{{\mathrm{T}}}}_1$, ${{{\mathrm{T}}}}_2$, …, ${{{\mathrm{T}}}}_{128}$). The total pulse widths a pixel may emit is $3 \times 8 = 24$, which incurs considerable time and energy.

**Fig. 6:**
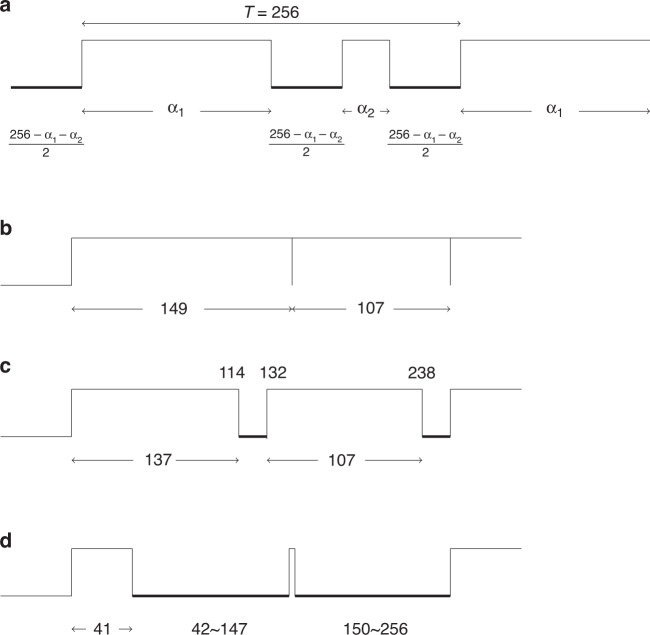
LCD PWM design for C_235_. **a** Generation of pulse widths in PWM. **b** For width 256 = 149 + 107, 2^off(0,0)^. **c** For width 220 = 113 + 107, 2^off(114~132, 238~256)^. **d** For width 44 = 41 + 3, 2^off(42~147,150~256)^

In contrast, in our case, based on Goldbach’s conjecture, for an integer between 6 and 254, *X* can be explored by C_235_ as

$$X = yT_y + z\,T_z$$

where $T_y$ and $T_z$ are binary variables and *y* and *z* are primes, $y,\,z \in \left\{ {3,\,5,\,7,\,11,\,13,\,17,\,19,\,23,\,29,\,31,\,37,\,41,\,43,\,47,\,53,\,58,\,61,\,67, \ldots ,139} \right\}$. With the specified 31 primes, C_235_ can generate all even numbers by adding the two closest prime numbers. Each prime represents a pulse width. Therefore, the LCD control center first computes the required prime value to generate a specific light for a pixel, and then orders the pixel circuits to control each of its R, G, and B LEDs to emit two pulse widths. The total pulse widths generated is $2 \times 3 = 6$, as shown in Fig. 6. Figure 6a shows that C_235_ can use two pulses only to generate any pulse between 0 and 256. Figure 6b shows that a width of 256 is generated by 149 and 107. Figure 6c, d illustrate that width 220 is composed of 113 and 107; and width 44 is composed of 41 and 3. Since the number of pulses required to generate in C_235_ LCD PWM is much less than that in the current LCD PWM, the required time and energy can be reduced significantly. This scheme could be valuable for designing high-rate future LCDs. Notably, 24 pulse widths are needed to generate a traditional PWM. With the C_235_ PWM, we can generate ${{{\mathrm{T}}}}_{131}$ and ${{{\mathrm{T}}}}_{113}$ for R; ${{{\mathrm{T}}}}_{112}$ twice for G, and ${{{\mathrm{T}}}}_{43}$ and ${{{\mathrm{T}}}}_{59}$ for B. In total, only six pulse widths are required.

An extended Goldbach conjecture can help us overcome the drawbacks of the current LCDs that demand many pulse widths for generating R, G, and B brightness. Goldbach’s conjecture states that any even number can be given as the sum of two prime numbers. From Goldbach’s conjecture, we have the following proposition.

<Use Goldbach Conjecture for expressing RGB>

Any even integer between 8 and 256 can be expressed as a sum of two elements in the following prime set *P* that contains all prime numbers between 3 and 149:

$$P = \left\{ {\begin{array}{*{20}{c}} {3,\,5,\,7,\,11,\,13,\,17,\,19,\,23,\,29,\,31,\,37,\,41,\,43,\,47,\,53,\,59,\,61,\,67,\,71,} \\ {79,\,83,\,89,\,97,\,101,\,103,\,107,\,109,\,113,\,127,\,131,\,139,\,149} \end{array}} \right\}$$

For instance, 256 = 149 + 107, 200 = 103 + 97, and 90 = 67 + 23.

It uses 34 primes to express any even number between 4 and 256. We are interested in forming a smaller prime set to express all numbers between 4 and 256 within a tolerable margin of error.

Suppose that there is a set of integers, expressed in the form of

$$n = 4 + 6k,\,{{{\mathrm{for}}}}\,k \in \left\{ {1,\,2,\,3,\, \ldots ,\,42} \right\}$$

In other words, *n* belongs to the integer set $N = \left\{ {10,\,16,\,22,\,28,\,34,\,40,\,46,\, \ldots ,\,244,\,250,\,256} \right\}$. We hope to find the minimal prime set $P_8^m$, where each of *n* in *N* can be expressed as the sum of two primes in $P_8^m$. This problem can be formulated as the following integer linear program^15,16^:

A program for finding $P_8^m$

$${{{\mathrm{Minimize}}}}\mathop {\sum }\limits_{q \in P} \delta _q = \delta _5 + \delta _7 + \delta _{11} + \delta _{13} + \cdots + \delta _{139} + \delta _{149}$$

subject to

$$10 = 10\delta _5$$

$$16 = 5\delta _5 + 7\delta _7 + 11\delta _{11} + 13\delta _{13}$$

$$22 = 5\delta _5 + 7\delta _7 + 11\delta _{11} + 13\delta _{13} + 17\delta _{17} + 19\delta _{19}$$

$$28 = 5\delta _5 + 7\delta _7 + 22\delta _{11} + 13\delta _{13} + 17\delta _{17} + 19\delta _{19} + 23\delta _{23}$$

$$34 = 5\delta _5 + 7\delta _7 + 11\delta _{11} + 13\delta _{13}, + 34\delta _{17} + 19\delta _{19} + 23\delta _{23} + 29\delta _{29} + 31\delta _{31}$$

$$40 = 5\delta _5 + 7\delta _7 + 11\delta _{11} + 13\delta _{13} + 17\delta _{17} + 19\delta _{19} + 23\delta _{23} + 29\delta _{29} + 31\delta _{31} + 37\delta _{37}$$

$$\vdots$$

$$256 = \mathop {\sum }\limits_{l \in P} l\delta _l,\,P = \left\{ {3,\,5,\,7,\, \ldots ,\,149} \right\}$$

where $\delta _k$ are binary variables for $k = 1,\,2,\,3,\, \ldots ,\,42$.

This program intends to minimize the number of primes in set *P*, subject to the restriction that all integers in *N* can be expressed as the sum of two primes. The above is a linear 0-1 program that can be efficiently solved by most commercial optimization software. The solution to the above program is

$\delta _k = 1$ for $k \in P_8^m = \left\{ {5,\,11,\,17,\,23,\,41,\,53,\,71,\,83,\,107,\,113,\,131,\,137,\,149} \right\}$, and $\delta _k = 0$ for $k \notin P^m$. The number of primes in set $P^m$ is 13, which is much smaller than the number of primes 34 in set *P*.

We then deduce the following proposition modified from Goldbach’s conjecture:

<Extended Goldbach conjecture for 8-bit lighting>

Consider any value *n* in an 8-bit RGB system. For $n = 4 + 6k,\,k \in \left\{ {1,\,2,\,3,\, \ldots ,\,42} \right\}$, the following propositions hold:

(i) Any value *n* can be expressed $n = p + q$, where $p,\,q \in P_8^m$.
(ii) Any value $n \pm 1,n \pm 2,n \pm 3$ can be approximately expressed as $n \pm \Delta \cong p + q$ for $\Delta = 1,\,2,\,3$ within an error rate of 3/*n*.
(iii) Prime *p* and *q* represent key color values in R, G, and B. Table 3 lists all $= 4 + 6k,\,k \in \left\{ {1,\,2,\,3,\, \ldots ,\,42} \right\}$ and the primes *p* and *q* satisfying *n* = *p* + *q*. For instance, 10 = 5 + 5, 40 = 23 + 17, 220 = 113 + 107,…, 256 = 149 + 107. It is noted that 220 can also be expressed as 149 + 71. Since 113 and 107 are closer to each other than 149 and 171, we adopt the expression 220 = 113 + 107. We can also approximately express 218, 219, 221, and 223 as the sum of 113 and 107 with an error within $\frac{3}{{220}} \cong 0.013$.

**Table 3:** Prime sets $P_8^m = \left\{ {5,\,11,\,17,\,23,\,41,\,53,\,71,\,83,\,107,\,113,\,131,\,137,\,149} \right\}$

*n*	*p* + *q*	*n*	*p* + *q*
10	5 + 5	136	83 + 53
16	11 + 5	142	101 + 41
22	11 + 11	148	107 + 41
28	17 + 11	154	83 + 71
34	17 + 17	160	107 + 53
40	23 + 17	166	83 + 83
46	41 + 5	172	131 + 41
52	41 + 11	178	107 + 71
58	41 + 17	184	113 + 71
64	41 + 23	190	107 + 83
70	53 + 17	196	113 + 83
76	71 + 5	202	131 + 71
82	41 + 41	208	137 + 71
88	47 + 41	214	107 + 107
94	53 + 41	220	113 + 107
100	83 + 17	226	113 + 113
106	53 + 53	232	131 + 101
112	71 + 41	238	131 + 107
118	101 + 17	244	131 + 113
124	71 + 53	250	149 + 101
130	113 + 17	256	149 + 107

Following the International Commission on Illustration, the tolerable rate of R, G, B brightness is 10%. Therefore, a brightness level larger than 30 is an admissible estimate within the tolerable error.

8-bit LCD PWM

A light color $2^\alpha 3^\beta 5^\sigma$ can be generated by assigning the two off-time periods for each of $\alpha ,\,\beta ,\,{{{\mathrm{and}}}}\,\sigma$ specified as follows.

$$2^{{{{\mathrm{off}}}}\left( {\alpha _1 + 1\sim \frac{{258 + \alpha _1 - \alpha _2}}{2},\,\frac{{256 + \alpha }}{2}\sim 256} \right)}$$

$$3^{{{{\mathrm{off}}}}\left( {\beta _1 + 1\sim \frac{{258 + \beta _1 - \beta _2}}{2},\,\frac{{256 + \beta }}{2}\sim 256} \right)}$$

$$5^{{{{\mathrm{off}}}}\left( {\sigma _1 + 1\sim \frac{{258 + \sigma _1 - \sigma _2}}{2},\,\frac{{256 + \sigma }}{2}\sim 256} \right)}$$

where $$\alpha _1 \ge \alpha _2,\,\beta _1 \ge \beta _2,\sigma _1 \ge \sigma _2,\,{{{\mathrm{for}}}}\,\alpha _1,\alpha _2,\,\beta _1,\beta _2,\sigma _1,\sigma _2 \in P_8^m$$, and $$\alpha _1 + \alpha _2 \cong \alpha ,\,\beta _1 + \beta _2 \cong \beta ,$$ $$\sigma _1 + \sigma _2 \cong \sigma$$.

The error of the generated light is smaller than $\frac{3}{\alpha },\,\frac{3}{\beta },$and $\frac{3}{\sigma }$ for R, G, and B lights, respectively.

## Materials and methods

### Colorizing objects

The world is full of multi-attribute objects^14,15^. If we colorize objects corresponding to attributes and allocate these colorized objects on a ring, we can visualize the corresponding relationships. By modifying a C_235_ wheel, we can design a C_235_ ring. Our method is also suitable for visualizing the relationship among them. By modifying a C_235_ color wheel, we can design a C_235_ ring for this purpose.

Consider a set of 16 people, identified as $\left\{ {a,\,b,\, \cdots ,\,p} \right\}$. Each person has a unique feature specified by three attributes, i.e., education, income, and age. Each of the three attributes has five values {0, 1, 2, 3, 4} indicating the status “not available”, “low”, “fair”, “middle”, and “high”, respectively. By assigning colors “cyan”, “magenta”, and “yellow” to education, income, and age, respectively, we associate each person *x* with a color code *Code*(*x*) and a number *Number*(*x*) as given in the following:

$$\begin{array}{ll}{\mathrm{Code}}\left( x \right) = \left\langle {2^{\alpha _x}3^{\beta _x}5^{\sigma _x}} \right\rangle ,\,{{{\mathrm{and}}}}\,{\mathrm{Number}}\left( x \right)\\ \qquad\qquad= \alpha _x + 5\beta _x + 25\sigma _x\,{{{\mathrm{for}}}}\,x \in \left\{ {a,\,b,\, \cdots ,\,p} \right\}\end{array}$$

**Fig. 7:**
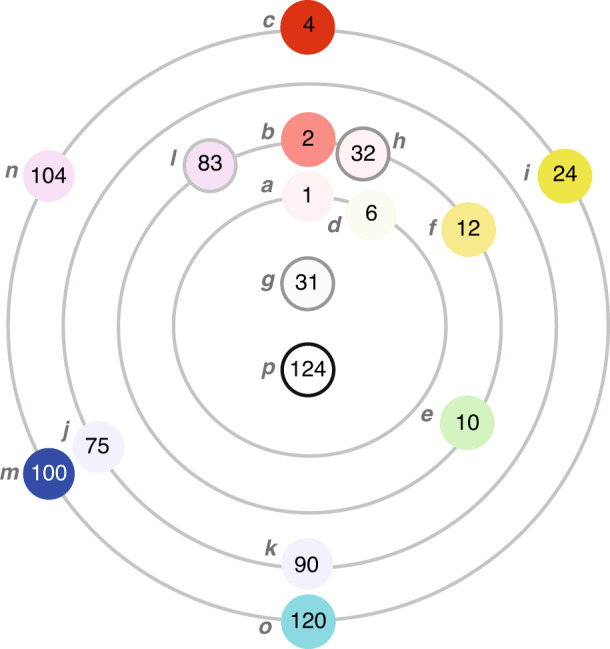
Visualization of the relationship of 16 people’s features. 16 people, identified as {a, b, …, p} are specified by three attributes, each has 5 category values. All these 16 individuals can be allocated on a 4-ring hue circle
(ii) Person *h* is numbered as 32 with color code $\left\langle {2^2 \times 3^1 \times 5^1} \right\rangle = \left\langle {2 \times 30} \right\rangle$, the mixture of very light red with very light gray, which is depicted as an inner small circle with very light red and an outer ring with very light gray.
(iii) Person *c* and person *d* are complementary with each other, where $\left\langle {2^4 \times 15^4} \right\rangle = \left\langle {30^4} \right\rangle$. Persons *i*, *n*, and *o* are triad-complementary because$\left\langle {4^4} \right\rangle \times \left\langle {10^4} \right\rangle \times \left\langle {15^4} \right\rangle = \left\langle {30^8} \right\rangle$.

(i) Then, we have the information conveyed in Table 4 and the relationship of the people in Fig. 7. All these 16 individuals can be allocated on a four-ring hue circle, which is used to visualize the relationship of these people’s features. Person *a* is represented by number 1, since $Number\left( a \right) = \alpha _a + 5\beta _a + 25\sigma _a$, where $\alpha _a = 1$ (low education) and $\beta _a = \sigma _a = 0$. Number 1 has color code $\left\langle {2^1} \right\rangle$, which is a red hue at level 1 (very light red). Similarly, person *c* ($\alpha _c = 4$, $\beta _c = \sigma _c = 0$) has the color codes $\left\langle {2^4} \right\rangle$ (red).

**Table 4:** Assigning color codes of individual people with multiple attributes

Person	Attribute	Number	Color Code	Color
*a*	low edu	1	$\left\langle {2^1} \right\rangle$	
*b*	mid edu	2	$\left\langle {2^2} \right\rangle$	
*c*	high edu	4	$\left\langle {2^4} \right\rangle$	
*d*	low edu, low income	6	$\left\langle {6^1} \right\rangle$	
*e*	fair income	10	$\left\langle {3^2} \right\rangle$	
*f*	fair edu, fair income	12	$\left\langle {6^2} \right\rangle$	
*g*	low edu, low income, low age	31	$\left\langle {30^1} \right\rangle$	
*h*	fair edu, low income, low age	32	$\left\langle {2^130^1} \right\rangle$	
*i*	high edu, high income	24	$\left\langle {6^4} \right\rangle$	
*j*	mid age	75	$\left\langle {5^3} \right\rangle$	
*k*	mid income, mid age	90	$\left\langle {15^3} \right\rangle$	
*l*	mid edu, low income, mid age	83	$\left\langle {2^25^230^1} \right\rangle$	
*m*	high age	100	$\left\langle {5^4} \right\rangle$	
*n*	high edu, high age	104	$\left\langle {10^4} \right\rangle$	
*o*	high income, high age	120	$\left\langle {15^4} \right\rangle$	
*p*	high edu, high income, high age	124	$\left\langle {30^4} \right\rangle$

### Colorizing DNA codons

More interestingly, our C_235_ method can be harnessed to colorize DNA codons^17,18^ for revealing the relationship between 22 amino acids and 64 genetic codons. There are 22 amino acids in a DNA codon, where each codon acid is composed of A, G, C, T^17^. For instance, acid 1 is composed of GCT, GCC, GCG, and GCA. By utilizing C_235_, we can assign each acid a color code and a number, and thus allocate all 22 amino acids on a C_235_ ring. If we denote the *a*_*l*_, *g*_*l*_, *c*_*l*_, and *t*_*l*_, the color codes of A, G, C, T at position *l* for *l* = 1, 2, 3, then we have

$$\begin{array}{*{20}{l}} {a_1 = < 2^13^2 > } \hfill & {a_2 = < 2^23^4 > } \hfill & {a_3 = < 2^33^6 > } \hfill \\ {g_1 = < 3^25^2 > } \hfill & {g_2 = < 3^45^4 > } \hfill & {g_3 = < 3^65^6 > } \hfill \\ {c_1 = < 2^2 > } \hfill & {c_2 = < 2^4 > } \hfill & {c_3 = < 2^6 > } \hfill \\ {t_1 = < 2^15^2 > } \hfill & {t_2 = < 2^25^4 > } \hfill & {t_3 = < 2^35^6 > } \hfill \end{array}$$

**Fig. 8:**
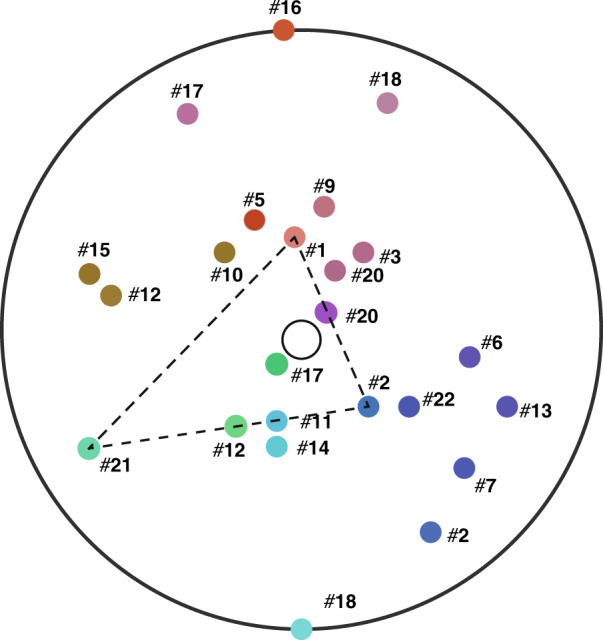
C_235_ ring of DNA codons. All 22 acids are displayed on a C_235_ ring. It is convenient to visualize the complementary and triad-complementary relations

Since T-A and C-G are complementary pairs, we let $t_l \times a_l = \left\langle {30^{2l}} \right\rangle$ and $c_l \times g = \left\langle {30^{2l}} \right\rangle$. The color codes of GCT in acid #1 is given as $g_1c_2t_3 = \left\langle {2^230^2(2^35^6)} \right\rangle = \left\langle {2^55^630^2} \right\rangle$. Similarly, the color codes of GCC, GCG, and GCA are

$$g_1c_2c_3 = \left\langle {2^830^2} \right\rangle ,\,g_1c_2g_3 = \left\langle {3^45^430^4} \right\rangle ,\,g_1c_2a_3 = \left\langle {2^33^230^2} \right\rangle$$

respectively. The color code of acid #1 is calculated as

$${\mathrm{Color}}\left( {{\mathrm{acid}}\,1} \right) = \left\langle {(2^230^2)^430^{12}} \right\rangle = \left\langle {2^830^{20}} \right\rangle$$

which can be illustrated as a small circle, where the inner circle has the color of 2^8^ (i.e., red at level 8) and the outer circle the color of 30^20^, i.e., gray at level 20 (Table 5). We can also assign a unique number to acid #1:

$${\mathrm{Number}}\left( {{\mathrm{acid}}\,1} \right) = \alpha _1 + 37\beta _1 + 37^2\sigma _1 = 28 + 37 \times 28 + 37^2 \times 28$$

where 37 is the total number of components of the 22 acids. All acids can be displayed on a C_235_ ring shown in Fig. 8. The details of coloring DNA codons are described in Supplementary A—Application to colorizing DNA codons. As shown in Table 5, C_235_ colorizes acids #1, #2, #6, #9, and #21 respectively as $\left\langle {2^830^{20}} \right\rangle$, $\left\langle {3^85^230^{20}} \right\rangle$, $\left\langle {2^53^{14}30^6} \right\rangle$, $\left\langle {2^{11}3^230^6} \right\rangle$, and $\left\langle {5^630^{20}} \right\rangle$. Since $\left\langle {2^830^{20}} \right\rangle \times \left\langle {3^85^230^{20}} \right\rangle \times \left\langle {5^630^{20}} \right\rangle = \left\langle {2^83^85^830^{60}} \right\rangle = \left\langle {30^{68}} \right\rangle$, #1, #2 and #21 are triad-complementary. Also $\left\langle {2^53^{14}30^6} \right\rangle \times \left\langle {2^{11}3^230^6} \right\rangle = \left\langle {2^{16}3^{16}30^{12}} \right\rangle$ means that #6 and #9 are complementary, as illustrated in Fig. 8.

**Table 5:** Assigning color codes to geno codons

Amino Acid#	Geno Codon	Geno Color	Acid Color Code	Number
#1 Ala	GCT	$\left\langle {2^55^630^2} \right\rangle$		28,148
	GCC	$\left\langle {2^830^2} \right\rangle$		
	GCG	$\left\langle {3^45^430^4} \right\rangle$		
	GCA	$\left\langle {2^53^630^2} \right\rangle$		
#2 Arg	CGT	$\left\langle {2^15^630^4} \right\rangle$		31,174
	#2' CGC	$\left\langle {2^430^4} \right\rangle$		
	CGG	$\left\langle {3^85^830^2} \right\rangle$		
	CGA	$\left\langle {2^13^630^4} \right\rangle$		
	#2” AGG	$\left\langle {3^{11}5^930^1} \right\rangle$		18,652
	AGA	$\left\langle {3^830^4} \right\rangle$		
_⋮_	_⋮_	_⋮_	_⋮_	_⋮_
#22 Stop	#22' TAG	$\left\langle {3^75^530^3} \right\rangle$		8663
	TAA	$\left\langle {2^43^830^2} \right\rangle$		
	#22” TGA	$\left\langle {3^65^830^4} \right\rangle$		1682

Other than DNA codons, the C_235_ can colorize many other objects. For instance, the World Customs Organization has developed an HS (harmonized system) to classify millions of items of merchandise worldwide^20^. The current HS classification is a six-digit code displayed on a large text table. By utilizing the C_235_ color system, we can assign colors to HS merchandise codes to help people easily recognize each of these goods.

## Supplementary information

Supplementary Information for Unifying Colors by Primes

## Data Availability

All data were available in the main text and the supplementary materials. Supplementary information accompanies the manuscript on the *Light: Science & Applications* website (http://www.nature.com/lsa).
