# Supplementary material for: Unifying colors by primes
Source: Light Sci Appl. 2023 Feb 1;12:32. doi: 10.1038/s41377-023-01073-x (PMC9889338; doi:10.1038/s41377-023-01073-x)
Supplement: Supplementary file 1 — Supplementary Information for Unifying Colors by Primes [file 41377_2023_1073_MOESM1_ESM.docx]

Supplementary Information for

**Unifying Colors by Primes**

Han-Lin Li^1^, Shu-Cherng Fang^2^, Bertrand M. T. Lin^3^, Way Kuo^4*^

^1^Department of Management Science, City University of Hong Kong, Hong Kong, China

^2^Department of Industrial and Systems Engineering, North Carolina State University, Raleigh, NC, 27695, USA

^3^Institute of Information Management, Yang Ming Chiao Tung University, Taiwan, China

^4^Hong Kong Institute for Advanced Study, City University of Hong Kong, Hong Kong, China

*Corresponding author: Way Kuo (email: way@cityu.edu.hk)

Supplementary A: Application for colorizing DNA codons

The rise of computational biology and genomics makes the DNA codon table for indicating the relationship between 22 amino acids and 64 genetic codon very useful^1,2^. The following example describes our ideas for colorizing DNA codons.

**Example A1**: There are 22 amino acids in a DNA codon, where each codon acid is composed of A, G, C, T. For instance, acid 1 is composed of GCT, GCC, GCG, and GCA. By utilizing C_235_, we can assign each acid a color code and a number, and thus allocate all 22 amino acids on a C_235_ ring. Denote the *a_l_*, *g_l_*, *c_l_*, and *t_l_*, the color codes of A, G, C, T at position *l* for *l* = 1, 2, 3. Then, we have

| *a*_1_ = <2^1^3^2^> | *a*_2_ = <2^2^3^4^> | *a*_3_ = <2^3^3^6^> |
| --- | --- | --- |
| *g*_1_ = <3^2^5^2^> | *g*_2_ = <3^4^5^4^> | *g*_3_ = <3^6^5^6^> |
| *c*_1_ = <2^2^> | *c*_2_ = <2^4^> | *c*_3_ = <2^6^> |
| *t*_1_ = <2^1^5^2^> | *t*_2_ = <2^2^5^4^> | *t*_3_ = <2^3^5^6^> |

Since T-A and C-G are complementary pairs, we let $t_{l}\times a_{l}=\left\langle{30}^{2l} \right\rangle$ and $c_{l}\times g=\left\langle{30}^{2l} \right\rangle$. The color codes of GCT in acid #1 is given as $g_{1}c_{2}t_{3}=\left\langle2^{2}{30}^{2}(2^{3}5^{6}) \right\rangle=\left\langle2^{5}5^{6}{30}^{2} \right\rangle$. Similarly, the color codes of GCC, GCG, and GCA are

$g_{1}c_{2}c_{3}=\left\langle2^{8}{30}^{2} \right\rangle, g_{1}c_{2}g_{3}=\left\langle3^{4}5^{4}{30}^{4} \right\rangle$, $g_{1}c_{2}a_{3}=\left\langle2^{3}3^{2}{30}^{2} \right\rangle$

respectively. The color code of acid #1 is calculated as

$$Color\left( acid 1 \right)= \left\langle\left( 2^{2}{30}^{2} \right)^{4}{30}^{12} \right\rangle=\left\langle2^{8}{30}^{20} \right\rangle$$

which can be illustrated as a small circle, where the inner circle has the color of 2^8^ (i.e., red at level 8) and the outer circle with the color of 30^20^, i.e., grey at level 20 (Fig. 5 in the main text). We can also assign a unique number to acid #1:

$$Number\left( acid 1 \right)= \alpha_{1}+37\beta_{1}+{37}^{2}\sigma_{1}=28+37\times28+{37}^{2}\times28$$

where 37 is the total number of components of the 22 acids. All acids can be displayed on a C_235_ ring shown in Fig. S2. From the ring, we can see that acids #1, #2, and #21 form a triad-complementary; acids #6 and #9 form a mirror of diagonals; and acids #18 and #19 form a mirror on neutral.

| Position →  Letter ↓ | 1 | 2 | 3 |
| --- | --- | --- | --- |
| *a_l_* | <2^1^3^2^> | <2^2^3^4^> | <2^3^3^6^> |
| *g_l_* | <3^2^5^2^> | <3^4^5^4^> | <3^6^5^6^> |
| *c_l_* | <2^2^> | <2^4^> | <2^6^> |
| *t_l_* | <2^1^5^2^> | <2^2^5^4^> | <2^3^5^6^> |


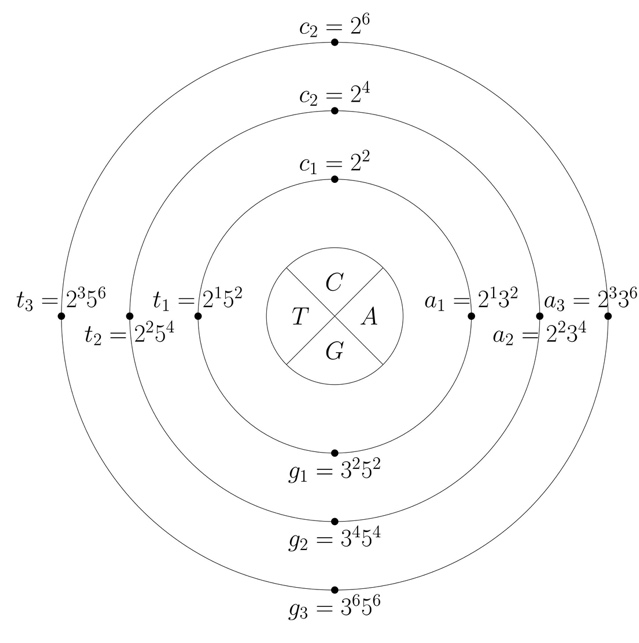


Fig. S1 Location of color codes.

Most genes are now discovered at the DNA level because of the development of genomics, and so a DNA codon table is becoming increasingly useful. A genetic codon table is shown in Fig. S1, where T and A, as well as C and G, complement each other. Table S1 shows a complete list of genetic codes which are aggregated into 22 amino acids, where the first acid is composed of GCT, GCC, GCG, GCA, denoted as

acid 1 = {GCT, GCC, GCG, GCA}

**Table S1 Color codes of DNA.**

**The number is computed according to** $\boldsymbol{l}_{\boldsymbol{i}}\boldsymbol{=}\boldsymbol{\alpha}_{\boldsymbol{i}}\boldsymbol{+37}\boldsymbol{\beta}_{\boldsymbol{i}}\boldsymbol{+}\boldsymbol{37}^{\boldsymbol{2}}\boldsymbol{\sigma}_{\boldsymbol{i}}$


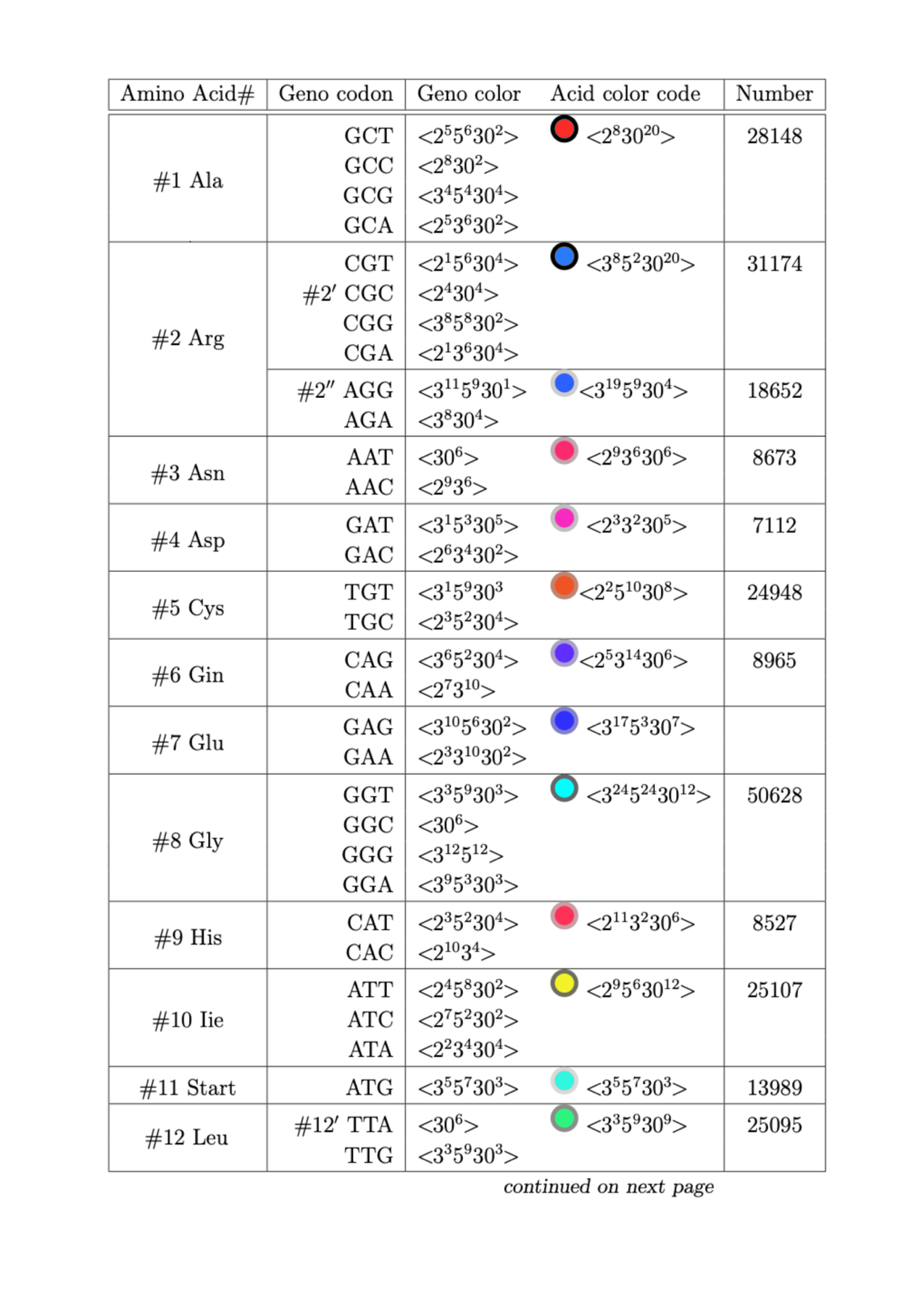


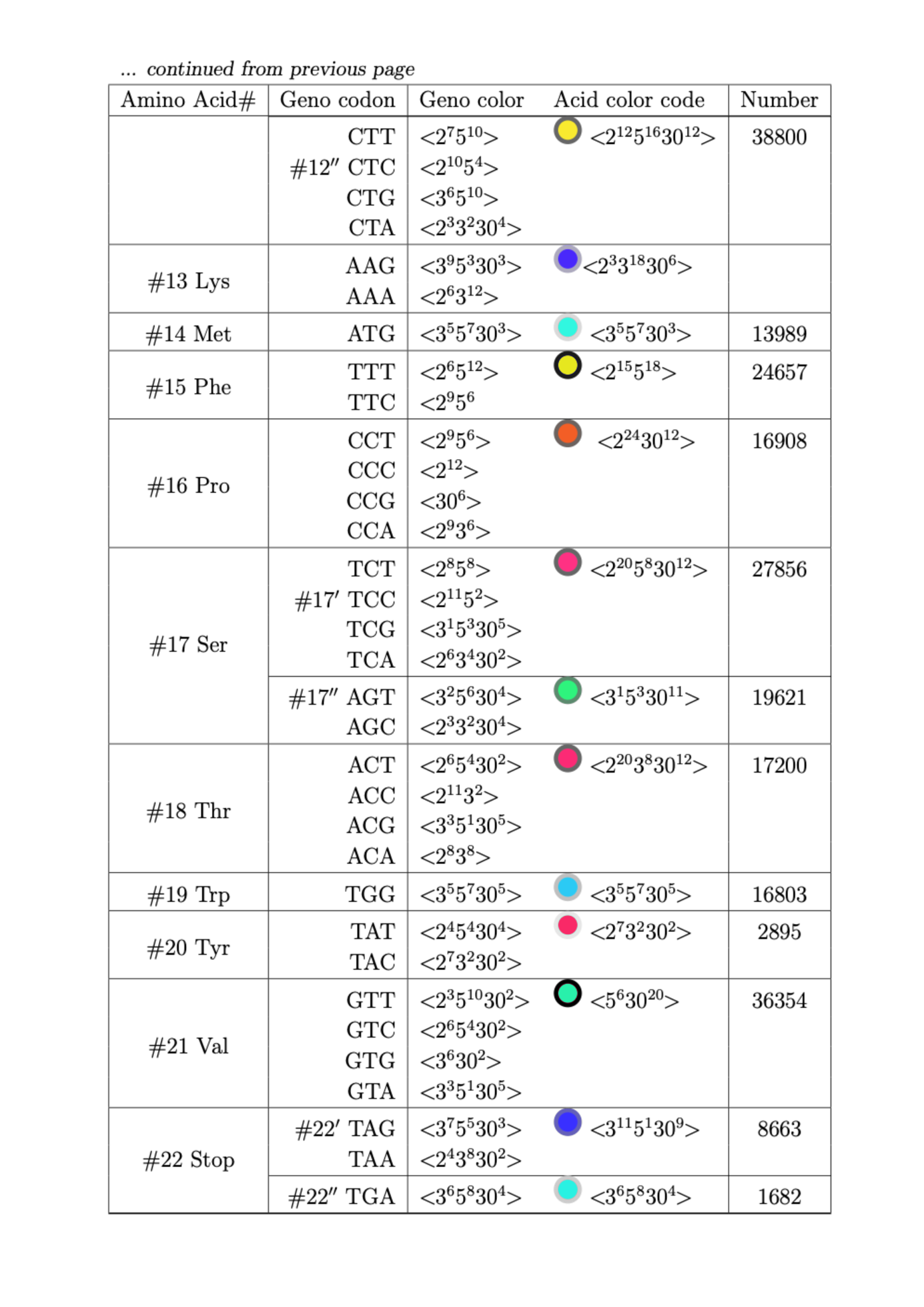


By utilizing C_235_, this study translates Table S1 into a color circle that is more useful for seeing the relationship among genetic codon and amino acids. We first specify a three-letter genetic code as

(*L*_1_, *L*_2_, *L*_3_) , where *L_i_* in {T, C, G, A} for *i* = 1, 2, 3

Using C_235_, we can assign each letter of {T, C, G, A} a color code. Denote *t_l_*, *c_l_*, *g_l_* and *a_l_* respectively the color codex of T, C, G, A at the position *l*. With reference to the hue circle, these color codes are assigned as shown on Fig. S1.

Since T-A, C-G are two complementary pairs, we let $t_{l}\times a_{l}=\left\langle{30}^{2l} \right\rangle$, $c_{l}\times g_{l}=\left\langle{30}^{2l} \right\rangle$, for *l* = 1,2,3, and we assign each DNA codon a color code. Consider four gene codes GCT, GCC, GCG, and GCA in amino acid 1 (Table S1) for instance. We compute the color for *g*_1_*c*_2_ as

$$g_{1}c_{2}=\left\langle3^{2}5^{2}2^{4} \right\rangle=\left\langle2^{2}{30}^{2} \right\rangle$$

Then, the color code of GCT becomes$g_{1}c_{2}t_{3}=\left\langle2^{2}{30}^{2}(2^{3}5^{6}) \right\rangle=\left\langle2^{5}5^{6}{30}^{2}) \right\rangle$.

Similarly, we compute the color codes of GCC, GCG and GCA as $g_{1}c_{1}c_{2}=\left\langle2^{2}{30}^{2}(2^{6}) \right\rangle=\left\langle2^{2}2^{6}{30}^{2}) \right\rangle$, $g_{1}c_{1}g_{3}=\left\langle2^{2}{30}^{2}(3^{6}5^{6}) \right\rangle=\left\langle3^{4}5^{4}{30}^{4}) \right\rangle$, and $g_{1}c_{1}a_{3}=\left\langle2^{2}{30}^{2}(2^{3}3^{6}) \right\rangle=\left\langle2^{4}3^{6}{30}^{2}) \right\rangle$, respectively.

For acid 1 in {GCT, GCC, GCG, GCA}, the color code for acid 1 is calculated as

$$Color\left( acid 1 \right)=(g_{1}c_{2})^{4}\left( t_{3}\times c_{3}\times g_{3}\times a_{3} \right)=\left\langle(2^{2}{30}^{2})^{4}\left. {30}^{12} \right\rangle\right.=\left\langle2^{8}{30}^{20} \right\rangle$$

The color of acid 1 can be illustrated as a small circle where the inner circle has the color of 2^8^ (i.e., cyan color at hue level of 8) and the outer circle has the color of <30^20^> (i.e., grey color at black-white level of 20). Following this process, each of the 22 acids in Table S1 has a unique color code.

We can also form a number set *N*(37) for all acids in Table S1, specified as *N*(37) = {28148, 31174, …, 1682}, where the number is assigned as *l_i_* = *a_i_* + 37*b_i_* +1369*c_i_*, as listed on the last column of Table S1. The table is converted into a colorful acid-geno map displayed on Fig. S2, which is plotted based on *HC*(24). For instance, acid 1 with color code $\left\langle2^{8}{30}^{20} \right\rangle$ is assigned on the top of ring 8. Fig. S2 illustrates some interesting relationships, as summarized in Table S2. These relationships are described in the following:

1. Acids #1, #2 and #21 form a triad complementary.
2. There are four complementary pairs.
3. Acids #6 and #9 form a “mirror of diagonal” relationship.
4. Acids #17 and #18 form a “mirror on neutral” relationship.
5. Acids #11 and #22 form approximately a monotonic.


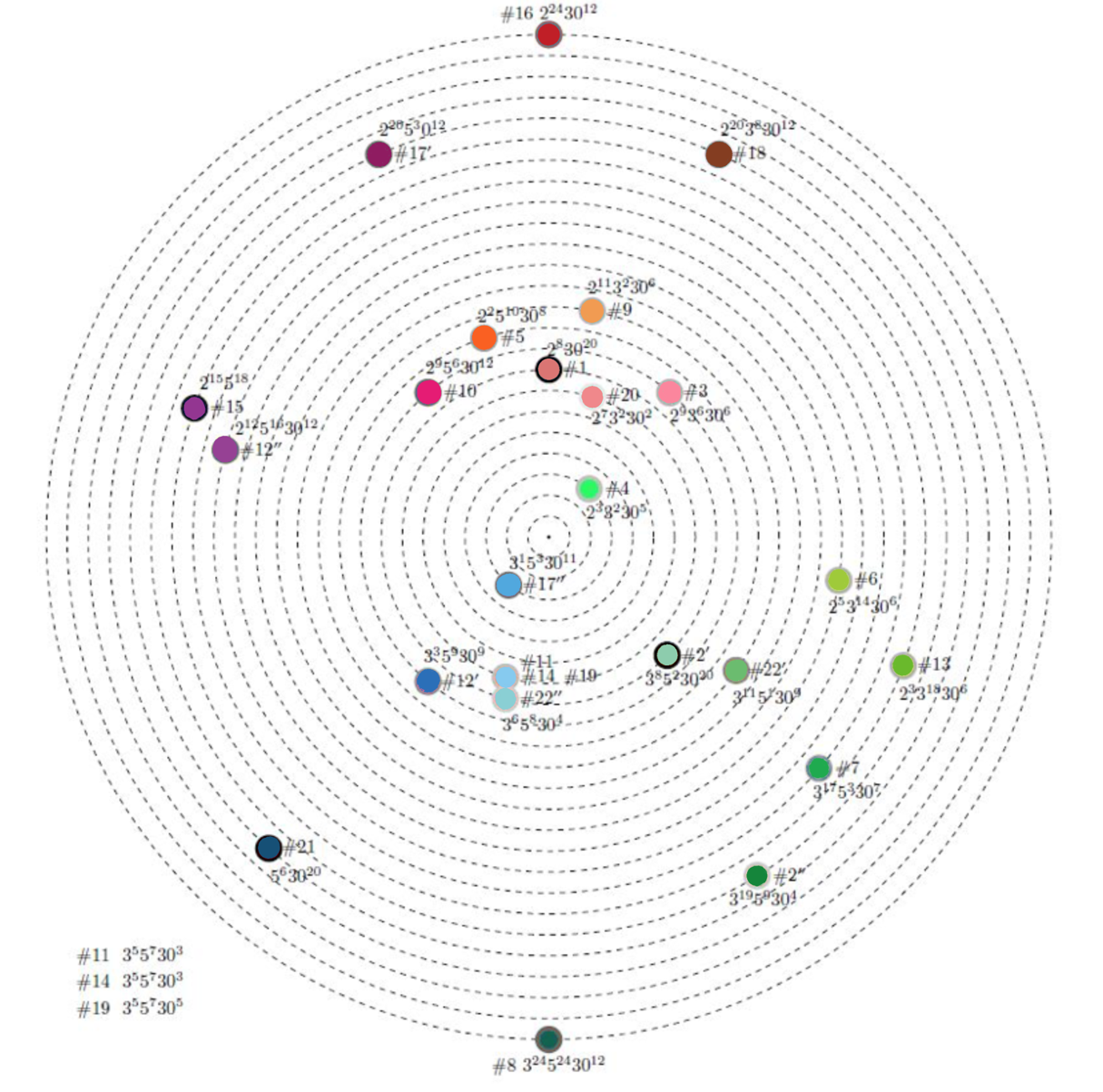


**Fig. S2 A C_235_ ring for DNA codons with detail codes.**

Table S2 Relationships among colorized acids in Table S1.

Our C_235_ system can also be applied to encoding protein-binding sites. Fig. S3 shows a C_235_ circle for the common sites of *E. coli* based on Li and Fu^1^. The C_235_ system can be applied to colorizing protein-binding sites, thus helping biologists to identify the DNA common sites at a glance. An example of searching protein binding sites, as discussed in Li and Fu^1^, is described as follows. Given an 18-letter sequence, each 105 positions long, where each position contains a letter from the set {T, C, G, A} to find a common site of length 16 with the pattern:

*L*_1_ *L*_2_ *L*_3_ *L*_4_ *L*_5_       *L*_6_ *L*_7_ *L*_8_ *L*_9_ *L*_10_

where *L_i_*,  ∈ {T, C, G, A} and empty boxes 's mean the positions of ignored letters. The letters of positions *L_i_* and $L_{11-i}$ are expected to be complementary, i.e. T-A and C-G (Fig. S4).

There are four common sites for the fumarate and nitrate reduction regulatory protein (FNR):

1. TGTTTAAACA
2. TGAAATTTCA
3. GTGAATTCAC
4. TGTGATCACA


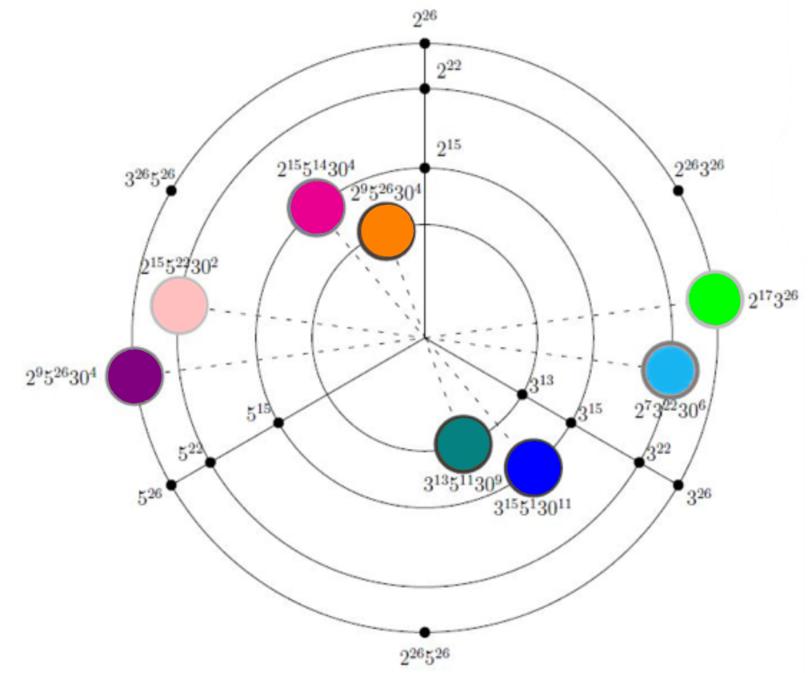


**Fig. S3 A C_235_ Ring for *E. coli* common sites.**

**Fig. S4 Codes for A-T-C-G positions.**

We can use the C_235_ system to assign each of these four common sites a unique color. Denote the color code of T, C, G, A at the position $L_{\mathcal{l}}$ as $t_{\mathcal{l}}$, $c_{\mathcal{l}}$, $g_{\mathcal{l}}$, and $a_{\mathcal{l}}$, respectively.

Then referring to Fig. S2, we let

$$\left. \begin{matrix} \begin{matrix} t_{\mathcal{l}}=\left\langle2^{\mathcal{l}}5^{2\mathcal{l}} \right\rangle\\ c_{\mathcal{l}}=\left\langle2^{2\mathcal{l}} \right\rangle\end{matrix} \\ \begin{matrix} g_{\mathcal{l}}=\left\langle3^{2\mathcal{l}}5^{2\mathcal{l}} \right\rangle\\ a_{\mathcal{l}}=\left\langle2^{\mathcal{l}}3^{2\mathcal{l}} \right\rangle\end{matrix} \end{matrix} \right\}, \mathrm{for}\mathcal{l=}1, 2, 3, 4, 5$$

Clearly,

$$\left. \begin{matrix} t_{\mathcal{l}}a_{\mathcal{l}}=\left\langle2^{2\mathcal{l}}3^{2\mathcal{l}}5^{2\mathcal{l}} \right\rangle=\left\langle{30}^{2\mathcal{l}} \right\rangle\\ c_{\mathcal{l}}g_{\mathcal{l}}=\left\langle2^{2\mathcal{l}}3^{2\mathcal{l}}5^{2\mathcal{l}} \right\rangle=\left\langle{30}^{2\mathcal{l}} \right\rangle\end{matrix} \right\}, \mathrm{for}\mathcal{l=}1, 2, 3, 4, 5$$

That means, since T-A are complementary, the merger of colors of $T_{\mathcal{l}}$ and $A_{\mathcal{l}}$ becomes “grey” at level $2\mathcal{l}$. Similarly, the merger of colors of $C_{\mathcal{l}}$ and $G_{\mathcal{l}}$ becomes “grey” at level $2\mathcal{l}$.

|  | *L*_1_, *L*_10_ | *L*_2_, *L*_9_ | *L*_3_, *L*_8_ | *L*_4_, *L*_7_ | *L*_5_, *L*_6_ |
| --- | --- | --- | --- | --- | --- |
| $a_{\mathcal{l}}$ | <2^1^3^2^> | <2^2^3^4^> | <2^3^3^6^> | <2^4^3^8^> | <2^5^3^10^> |
| $g_{\mathcal{l}}$ | <3^2^5^2^> | <3^4^5^4^> | <3^6^5^6^> | <3^8^5^8^> | <3^10^5^10^> |
| $c_{\mathcal{l}}$ | <2^2^> | <2^4^> | <2^6^> | <2^8^> | <2^10^> |
| $t_{\mathcal{l}}$ | <2^1^5^2^> | <2^2^5^4^> | <2^3^5^6^> | <2^4^5^8^> | <2^5^5^10^> |

Now consider the common site TGTTT-AAACA. We colorize TGTTT as

| *Color*(TGTTT) | = | *t*_1_ *g*_2_ *t*_3_ *t*_4_ *t*_5_ |
| --- | --- | --- |
|  | = | <2^1^5^2^> <3^4^5^4^> <2^3^5^6^> <2^4^5^8^> <2^5^5^14^> |
|  | = | <2^13^3^4^5^30^> |
|  | = | <2^9^5^26^30^4^> |

Similarly, AAACA is colorized as

| *Color*(AAACA) | = | *a*_1_ *a*_2_ *a*_3_ *c*_4_ *a*_5_ |
| --- | --- | --- |
|  | = | <2^1^3^2^> <2^4^> <2^3^3^6^> <2^4^3^8^> <2^5^3^10^> |
|  | = | <2^17^3^26^> |

The merger of the above two colors becomes

|  |  | *Color*(TGTTT) merge *Color*(AAACA) |
| --- | --- | --- |
|  | = | *t*_1_ *g*_2_ *t*_3_ *t*_4_ *t*_5_ × *a*_1_ *a*_2_ *a*_3_ *c*_4_ *a*_5_ |
|  | = | <2^9^5^26^30^4^> × <2^17^3^26^> |
|  | = | <30^30^>. |

A list of four colorized FNR *E. coli* common sites is illustrated in Table S3. The distribution of these four common sites of *E. coli* on a 26-ring C_235_ Ring is illustrated on Fig. S3.

Table S3 Color codes for four common sites of *E. coli*


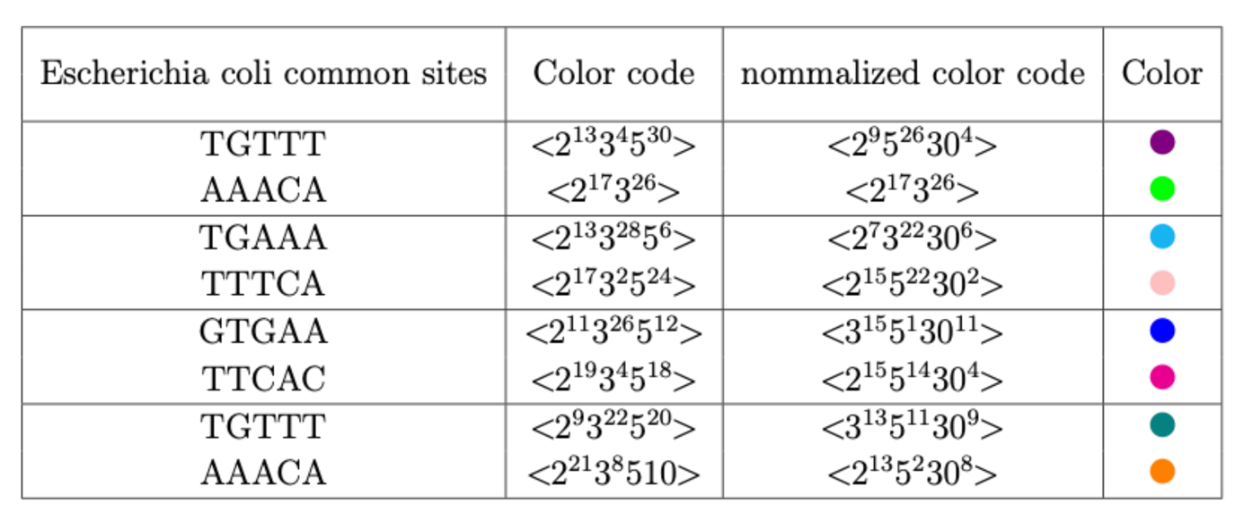


Supplementary B: Current LCD PWM

In LCDs that use PWM, the backlight is always on at its fullest brightness. If we want to achieve a lower brightness, then we need to turn the display on and off at a very high frequency. This frequency is not perceived by human eyes, if the flickers are faster than 60Hz (60 times per second) as consistent. However, the current LCD PWM has serious drawbacks described below.

For a 9-bit RGB LCD system, where each of R, G, and B has 2^9^ = 512 levels of brightness, the current PWM needs to generate 3×9 = 27 pulse widths or duty cycles for each pixel on an LCD screen. This mechanism incurs a considerable amount of energy and devices.

In a 8-bit RGB system, it requires 3×256 key values to express a mixed color. For a 9-bit RGB system, the number of key values becomes 3×512, which is a large number. The Goldbach Conjecture is one of the most essential theories to describe relations among integers. Following the Goldbach Conjecture, expressing 8-bit colors needs only 3×33 key values, and for express 9-bit colors requires only 3×56 key values.

In order to emit R, G and B lights, a pixel on an LCD screen has three LEDs under the control of a pixel circuit, where all pixel circuits follow the order of a source driver IC to control the brightness level and a gate driver IC to control the on-off state. For most of the current 9-bit display systems, the brightness levels of each of the R, G, B fall within [0, 512] and is expressed in the following way:

$$x=512w_{512}+128w_{128}+64w_{64}+32w_{32}+16w_{16}$$

$+8w_{8}+4w_{4}+2w_{2}+w_{1}$ ...Expression (B1)

where $w_{512},w_{128},w_{64},w_{32},w_{16},w_{8},w_{4},w_{2},w_{1}$ are binary variables.

The composition scheme indicates that the current source driver IC uses voltage to order each of the pixel current to generate up to 9 pulse widths for each of R, G, and B LEDs. In total, there are up to 27 pulse widths generated by pixel currents for emitting a specific light shows that LCD has two devices, a source driver and a gate driver, for controlling the pixel IC. For an 8-bit color system, there are 9 output pulse widths for each of R, G, and B. A 100% duty cycle provides 100% brightness. If we want to achieve a 50% brightness, it runs in a 50% duty cycle, "ON" for a half of the time and "OFF" for another half of the time. Similarly, if we want to achieve a 25% brightness, it runs in a 25% duty cycle, "ON" for one-fourth of the time and "OFF" for three-fourth of the time.

Fig. S5 illustrates that for a 9-bit LCD display, for each of R, G, and B lights, there are up to four off-time periods required to be set for each pixel. The more off-time periods generated, the more power energy and flickering incurred. Fig. S5a shows a current PWM system that consists of 9 pulse widths, Q_1_, Q_2_, ..., Q_9_. By referencing to Expression (B1), the widths of Q_1_, Q_2_, ..., Q_9_ are 256, 128, 64, ..., and 1. Fig. S5b shows that there is no off-time period for a 100% duty cycle, since $512=256+128+64+32+16+8+4+2+1$. Fig. S5c shows a duty cycle of $\frac{340}{512}\cong66\%$ based on the composition

$$340=256\times1+128\times0+64\times1+32\times0+16\times1+8\times0+4\times1+2\times0$$

There are 4 off-time periods, namely, 257~384, 449~480, 497~506, and 511~512.

Fig. S5d shows a duty cycle of $\frac{172}{512}\cong14\%$ based on the composition

$$340=256\times0+128\times1+64\times0+32\times1+16\times0+8\times1+4\times1+2\times0$$

There are 4 off-time periods, namely, 1~256, 385~448, 481~496, and 511~512.


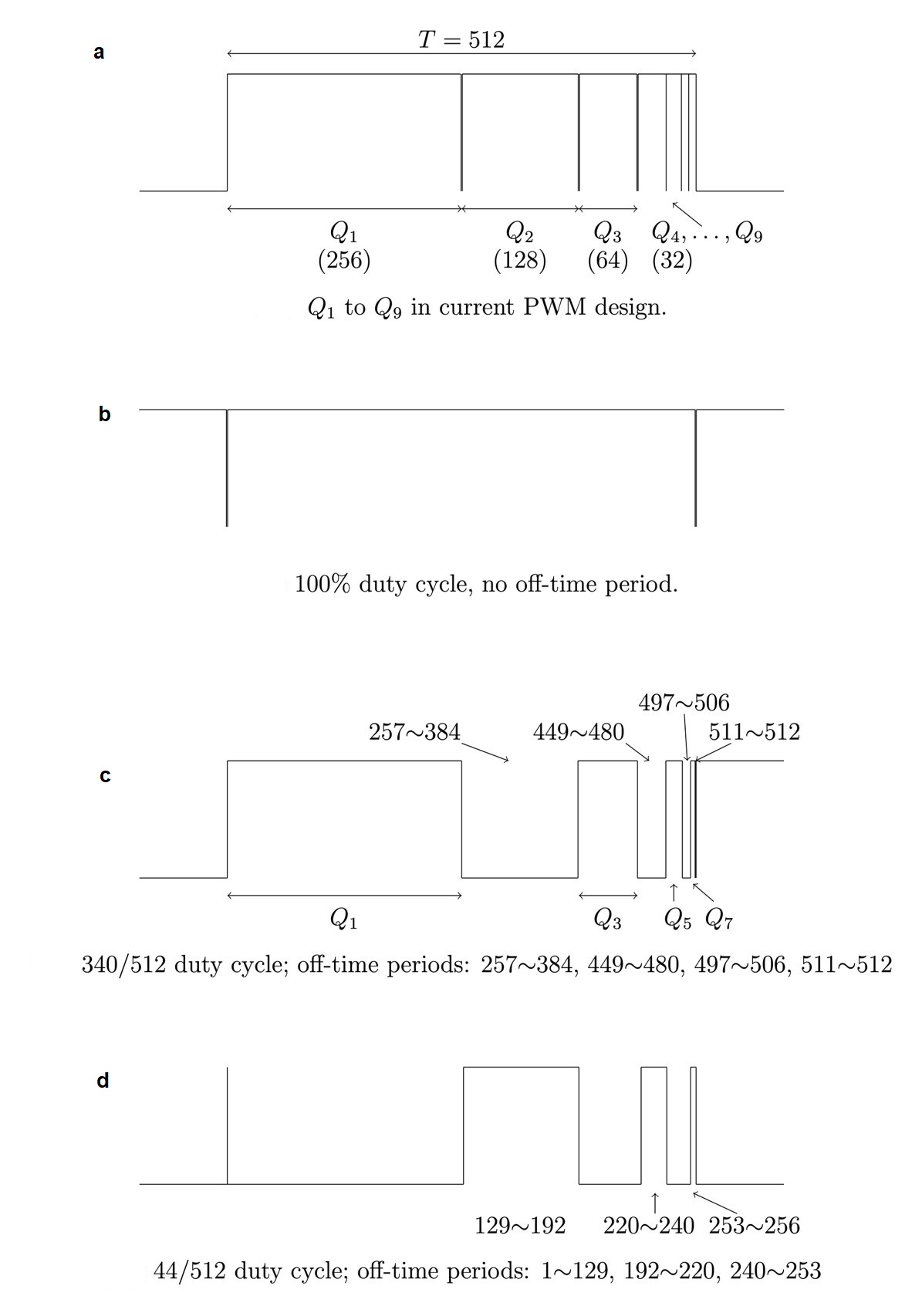


**Fig. S5 Pulse patterns for different duty cycles in current PWM.**

**References**

1. Li, H. L. & Fu, C. J., A linear programming approach for identifying a consensus sequence on DNA sequences. *Bioinformatics*. **21,** 1838-1845 (2005).
2. Shu, J. J. A new integrated symmetrical table of genetic codes, *Biosystems.* **151,** 21-26 (2017).
